# Assessing the Mass Concentration of Microplastics and Nanoplastics in Wastewater Treatment Plants by Pyrolysis Gas Chromatography–Mass Spectrometry

**DOI:** 10.1021/acs.est.2c07810

**Published:** 2023-02-14

**Authors:** Yanghui Xu, Qin Ou, Xintu Wang, Feng Hou, Peng Li, Jan Peter van der Hoek, Gang Liu

**Affiliations:** † Key Laboratory of Drinking Water Science and Technology, Research Centre for Eco-Environmental Sciences, Chinese Academy of Sciences, Beijing 100085, P. R. China; ‡ Section of Sanitary Engineering, Department of Water Management, Faculty of Civil Engineering and Geosciences, Delft University of Technology, Stevinweg 1, 2628 CN Delft, The Netherlands; § College of Environmental Science and Engineering, Guilin University of Technology, Guilin, Guangxi Province 541004, P.R. China; ∥ China Water Environmental Group Limited, Jinbao Street 89, 101101 Beijing, P.R. China; ⊥ Department Research & Innovation, Waternet, P.O. Box 94370, 1090 GJ Amsterdam, The Netherlands; # University of Chinese Academy of Sciences, Beijing 101408, P.R. China

**Keywords:** microplastics, nanoplastics, mass concentration, WWTPs, Py-GC/MS

## Abstract

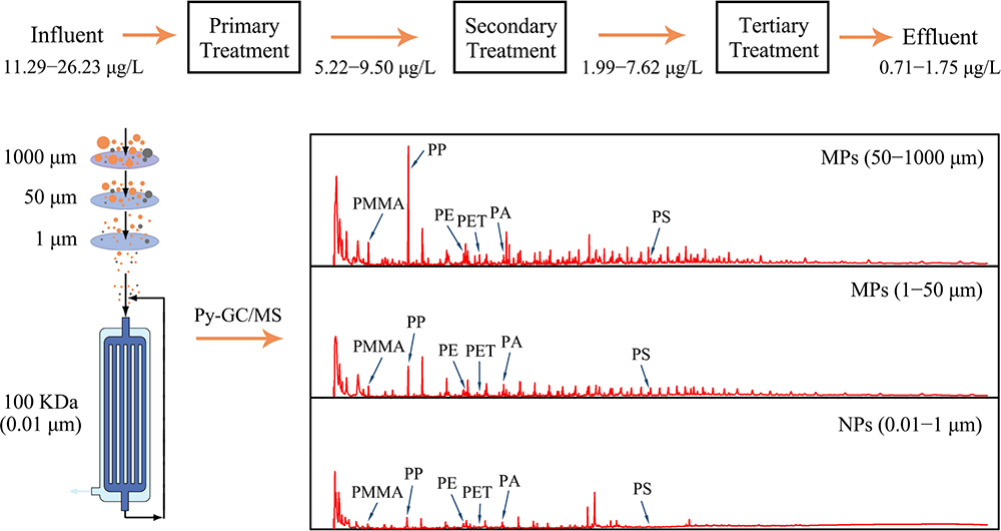

The level of microplastics (MPs) in wastewater treatment plants (WWTPs) has been well evaluated by the particle number, while the mass concentration of MPs and especially nanoplastics (NPs) remains unclear. In this study, pyrolysis gas chromatography–mass spectrometry was used to determine the mass concentrations of MPs and NPs with different size ranges (0.01–1, 1–50, and 50–1000 μm) across the whole treatment schemes in two WWTPs. The mass concentrations of total MPs and NPs decreased from 26.23 and 11.28 μg/L in the influent to 1.75 and 0.71 μg/L in the effluent, with removal rates of 93.3 and 93.7% in plants A and B, respectively. The proportions of NPs (0.01–1 μm) were 12.0–17.9 and 5.6–19.5% in plants A and B, respectively, and the removal efficiency of NPs was lower than that of MPs (>1 μm). Based on annual wastewater effluent discharge, it is estimated that about 0.321 and 0.052 tons of MPs and NPs were released into the river each year. Overall, this study investigated the mass concentration of MPs and NPs with a wide size range of 0.01–1000 μm in wastewater, which provided valuable information regarding the pollution level and distribution characteristics of MPs, especially NPs, in WWTPs.

## Abstract 2

This study assessed the mass concentration of MPs and NPs with a wide size range of 0.01−1000 μm in wastewater with Py-GC/MS. It revealed pollution levels and removal characteristics of MPs and NPs in WWTPs.

## Introduction

In recent years, the issue of small plastic particles known as microplastics (MPs) has been attracting increasing attention.^1,2^ MPs are plastics with a size below 5 mm, and the plastic particles smaller than 1 μm are referred to as nanoplastics (NPs).^3^ MPs were widely found in various environments such as seawater,^4,5^ freshwater,^6^ sediments,^7^ and soil.^8^ Studies reported that MPs can cause potential adverse effects on aquatic organisms,^9,10^ ecosystems,^11,12^ and human health.^13^ Many anthropogenic sources of MPs in the environment were reported, and municipal wastewater treatment plants (WWTPs) were considered as important urban sources releasing MPs.^14^ MPs like microbeads used in facial scrubs, toothpaste, and other personal care products can, through the sewer, reach WWTPs, where they may pass the wastewater treatment processes due to their small size.^15^ Although MPs were reported to be effectively removed during particulate matter removal in WWTPs, a considerable number of MPs can enter the aquatic environment due to the high flux of wastewater.^16^

The pollution level of MPs in WWTPs has been well studied in terms of the particle number concentration, which ranged from 1 to 18,285 particles/L in the influent and 0–447 particles/L in the effluent.^14,17^ Numerous studies have reported that WWTPs can effectively remove MPs from raw wastewater through primary, secondary, and tertiary treatment processes;^14^ the removal efficiency in different WWTPs was dependent on the treatment processes utilized.^18^ Generally, more than 88% of MPs could be removed by secondary treatment and over 97% of MPs could be removed when tertiary treatment was applied, such as a membrane bioreactor or rapid sand filtration technologies.^19,20^ However, the obtained knowledge on the level, removal, and impact of MPs in WWTPs was mostly based on the particle number rather than the mass. Compared with the mass, the particle number cannot well describe the pollution extent of MPs because their sizes can span a range of 3 orders of magnitude. In addition, MPs could be fragmented after physical and chemical processes,^21^ which may affect the number of particles and lead to a potential overestimation of their concentration.

Despite the apparent advantages of using mass to quantify the pollution level of MPs, limited studies have described the mass concentration of MPs in WWTPs. Simon et al. calculated the mass concentration of MPs based on the identification results from Fourier transform infrared spectroscopy (FTIR), the volume of the particle, and the polymer density.^17^ They reported that 61–1189 and 0.5–11.9 μg/L MPs were found in the influent and effluent of Denish WWTPs, respectively.^17,22^ However, it was an indirect and rough estimation of the mass of MPs in WWTPs. Thermal analytical methods, such as thermal extraction desorption–gas chromatography/mass spectrometry (TED-GC/MS) and pyrolysis gas chromatography mass spectrometry (Py-GC/MS), can accurately determine the mass of MPs,^23−26^ which may be workable for better understanding the extent of MP pollution in WWTPs compared with particle-related characterization. Using TED-GC/MS, Goedecke et al. reported that the mass concentration of MPs in the effluent of a WWTP ranged from 6.5 up to 51.8 μg/L.^22^ Although this study provides important data reference on masses of MP emissions in WWTPs, data on the mass concentration, especially the mass removal efficiency of MPs, are still very limited.

Quantification of MPs with a small size in WWTPs is another knowledge gap. As reported, the minimum particle size of MPs in WWTPs studied in previous articles was usually 20 or 50 μm.^19,27−29^ The pollution level of small-size MPs, especially NPs, with a size smaller than 1 μm, remains unclear. FTIR and Raman microspectroscopy, the most frequently used methods, can identify MPs with sizes only down to approximately 20 and 1 μm, respectively, due to the limited spatial resolution and sample fluorescence interference.^30−32^ Recently, optical-photothermal infrared (O-PTIR) microspectroscopy was successfully used to identify small MPs and NPs down to 600 nm released from silicone–rubber baby teats.^33^ However, besides its limitations in determining NPs at smaller sizes (<600 nm), this technique is unlikely to detect NPs in complex wastewater environments due to interference of the water matrix.^34^ As mentioned above, the thermal analytical methods that quantify MPs by the mass concentration are not limited by particle size and may be a promising technique to detect NPs in WWTPs.

The objective of this study is to investigate the mass concentration of MPs and NPs in WWTPs by Py-GC/MS. An ultrafiltration-based method was further developed to concentrate and detect trace NPs in WWTPs. The influent and treated wastewater after primary, secondary, and tertiary treatment in two WWTPs in China were sampled. MPs and NPs in the size range of 0.01–1000 μm were extracted and divided into three groups with sieving sizes of 50–1000, 1–50, and 0.01–1 μm. Six polymer types including polymethyl methacrylate (PMMA), polypropylene (PP), polystyrene (PS), polyethylene (PE), polyethylene terephthalate (PET), and polyamide (PA) that are widely found in WWTPs were selected to assess the mean mass concentration of these MPs and NPs and mean removal efficiency of WWTPs.

## Materials and Methods

### Materials

Sodium iodide (CAS 7681-82-5), dichloromethane (CAS 75-09-2), and methanol (CAS 67-56-1) were purchased from Macklin Biochemical Co., Ltd. (Shanghai, China). PS NPs with a nominal size of 200 nm were purchased from Beijing Zhongkeleiming Technology Co., Ltd. (Beijing, China). PVC (CAS 9002-86-2), PMMA (CAS 9011-14-7), PP (CAS 9003-07-0), PS (CAS 9003-53-6), PE (CAS 9002-88-4), PET (CAS 25038-59-9), and PA (CAS 63428-83-1) were purchased from Macklin Biochemical Co., Ltd. (Shanghai, China). The polymer granules were frozen with liquid nitrogen, milled with a grinder for 30 min, and separated with 50, 100, and 500 mesh stainless steel sieves to harvest fine polymer powders with a size of 50–100 mesh and less than 500 mesh. Stainless steel membranes (1000, 50, and 1 μm) were purchased from Shuangte Filter Equipment Co. (Hebei, China). The polymer powders were cleaned with methanol several times, filtered with stainless steel membranes, and then dried in an oven at 65 °C. The purpose of cleaning with methanol is to remove possible dissolved organic matters, which had no significant effect on the surface morphology and size of these powders (Figure S1). These polymer powders were used for calibration curves and recovery determination. When preparing polymer powders with low calibration concentrations, it is difficult to weigh them directly. To solve this problem, the polymer powder was dispersed in a mixture of methylene chloride and methanol to facilitate the weighing of small amounts of polymer. The stock solution (10 g/L) was continuously diluted to obtain a plastic dispersion of 2–1000 mg/L, as described in Text S1.

### Sampling

**Figure 1:**
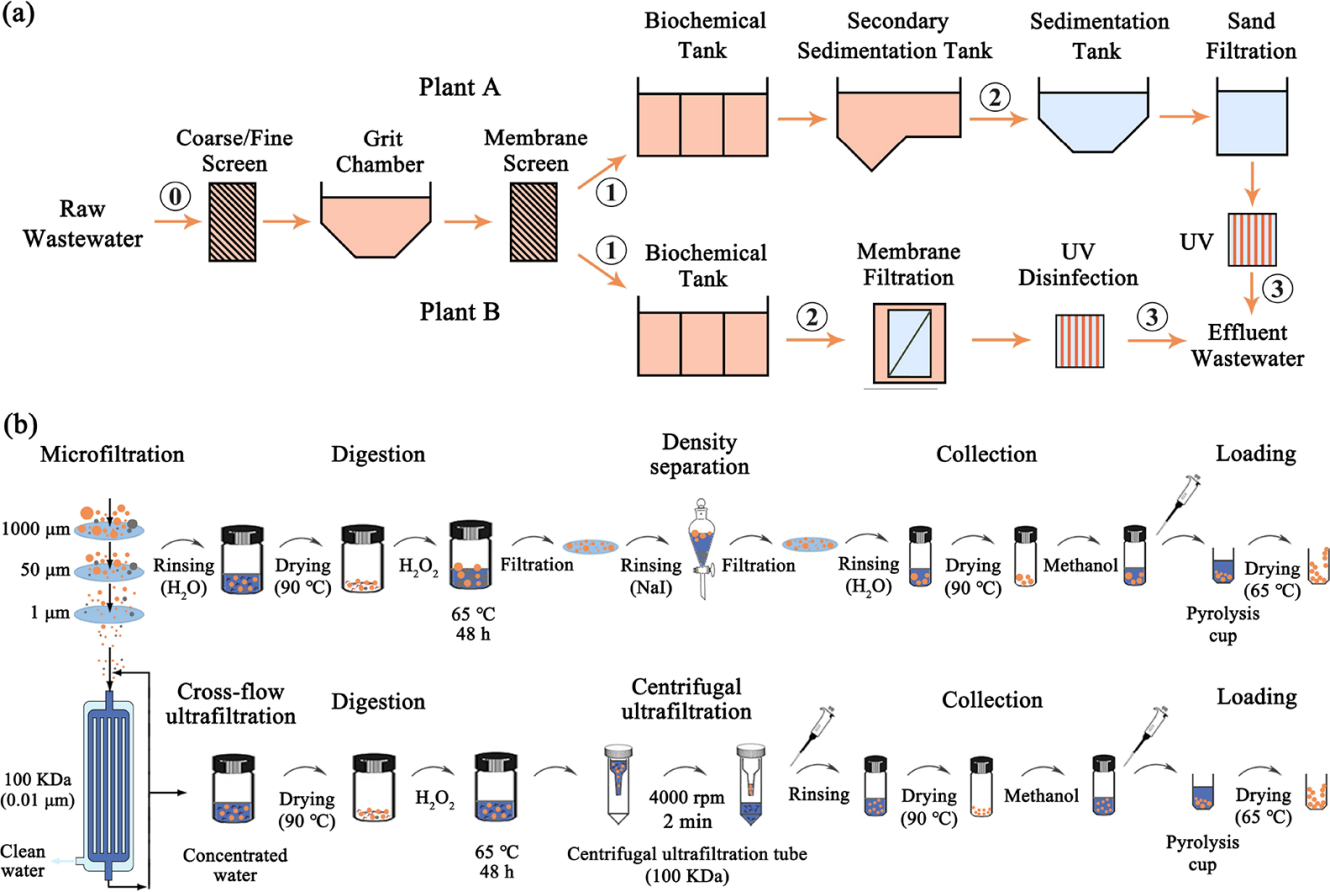
Flow chart of treatment processes and sampling sites in two WWTPs (a) and pretreatment procedures of wastewater samples for MP and NP detection (b). Sampling sites 0, 1, 2, and 3 mean raw wastewater and treated water after the primary, secondary, and tertiary treatment, respectively.

Flow charts of the treatment processes and sampling sites in two tested WWTPs in China (plants A and B) are shown in Figure 1a. Raw wastewater (0) and treated wastewater after the primary (1), secondary (2), and tertiary treatment (3) were collected from two WWTPs. The average inlet flow rates of plants A and B were about 5 × 10^5^ and 2 × 10^5^ m^3^/day, respectively, with the same values as the outlet flow rate. Raw wastewater of both plants was mainly from households. The coarse screen, fine screen, grit chamber, and membrane screen were the primary treatment steps of both plants; the secondary activated sludge tank of both plants was based on A2O technologies, but plant A contained a secondary sedimentation tank. The rapid sand filtration and UV disinfection were used for tertiary treatment in plant A; the tertiary treatment in plant B included a membrane bioreactor and UV disinfection (Figure 1a). Before sampling, the airtight plastic storage bucket and sampling bucket were washed several times with ultrapure water to avoid possible plastic contamination. At each sampling site, wastewater was picked up using a sampling bucket with a rope and put into prepared plastic buckets. In both WWTPs, 25 L of raw wastewater and 25, 50 L, and 100 L of treated wastewater after primary, secondary, and tertiary treatment, respectively, were collected and delivered to the laboratory within 1 day.

### Extraction and Pretreatment of MPs and NPs

As shown in Figure 1b, microfiltration, ultrafiltration, and hydrogen peroxide digestion were used to extract MPs and NPs from wastewater.^35^ To harvest MPs, the collected wastewater was first filtered by 1000, 50, and 1 μm stainless steel membranes. Macroscopic MPs larger than 1000 μm were not considered in this study because of their low count and the fact that certain large particles could not fit into the pyrolysis cup. MPs with sizes of 50–1000 and 1–50 μm were harvested from the membranes, transferred to a clean 50 mL glass bottle by ultrasound, and rinsed with ultrapure water. MP samples and concentrated filtered water samples containing NPs (0.01–1 μm) were dried at 90 °C in an oven. The solvent evaporation with 90 °C heating aimed to shorten the pre-treatment time,^19,36^ and this procedure had no significant effect on the surface morphology, size, and mass of selected polymers (Table S2 and Figure S1). The bottle was covered with aluminum foil to minimize possible contamination. Hydrogen peroxide (10 mL, 30%) was added to the dried MP samples in bottles and heated at 65 °C for 48 h on a stirring hotplate.^37^ After digestion, the suspension containing MPs larger than 1 μm was vacuum-filtered onto the stainless steel membranes. The membranes were sonicated and cleaned with 40 mL of saturated sodium iodide solution, and the suspension was transferred to a 100 mL separation funnel for density separation.^38^ After 12 h, the supernatant liquid was vacuum-filtered onto the stainless steel membranes, cleaned with ultrapure water, and then dried at 90 °C. Dried MP samples were sonicated and rinsed with methanol to transfer MPs in methanol. The resulting particle–methanol suspension was transferred gradually into a 10 mL glass bottle and evaporated at 65 °C to adjust the final volume to 1–2 mL. The final suspension was subsequently transferred to an 80 μL pyrolysis cup and dried at 60 °C in an oven to ensure that all MPs were loaded for subsequent detection by Py-GC/MS.

To harvest NPs, wastewater filtered with 1 μm stainless steel membranes was further concentrated using a cross-flow ultrafiltration (100 KDa, approximately 10 nm) system. The detailed steps for cross-flow ultrafiltration can be found in Text S2. The first step membrane filtration (>1 μm) may filter out NPs that were aggregated or adsorbed on large particles like MPs in wastewater, and these NPs were excluded from the downstream analysis. The concentrated suspension containing NPs was dried at 90 °C in an oven and then digested with 10 mL of 30% hydrogen peroxide (65 °C, 48 h). The suspension was put into a centrifugal ultrafiltration tube (15 mL) with a cutoff of 100 KDa and centrifuged at 4000 rpm for 2 min. The retentate was transferred into a 10 mL glass bottle. To minimize sample loss, 2 mL of ultrapure water was injected into the centrifugal ultrafiltration tube, and then, the membrane surface was gently blown with a pipetting tip. This step was repeated three times, and the washing liquid was also transferred into the glass bottle. The collected retentate was dried at 90 °C in an oven. Following the same procedure as for MP loading, dried NP samples were dispersed in methanol and eventually transferred into 80 μL pyrolysis cups for Py-GC/MS measurement. Two duplicates for each MP and NP sample were prepared.

### Pyrolysis Gas Chromatography–Mass Spectrometry

Pyrolysis GC/MS measurements were performed by a multi-shot pyrolyzer EGA/PY-3030D (Frontier Laboratories, Saikon, Japan) that was attached to an Agilent 7890A gas chromatograph (Santa Clara, CA) equipped with an HP-5MS column linked to an Agilent 5975C mass-spectrometer detector. Pyrolysis was performed according to the parameters used in previous studies.^24,39^ Briefly, pyrolysis temperature in single-shot mode was set at 650 °C for 0.2 min, and the interface temperature was set at 320 °C. The pyrolysis product was injected with a split ratio of 50:1. Additional details on the single-shot Py-GC/MS conditions can be found in Table S3. Mass-based concentrations were calculated by fitting the obtained results onto calibration curves.

Seven of the most commonly used plastic polymers including PVC, PMMA, PP, PS, PE, PET, and PA were analyzed to determine the characteristic indicator ions (Text S4 and Figure S2).^24,40−42^ The selectivity of the indicator ions was tested by analyzing several selected organic substances including wood, leaf, fish, humic acid, and black carbon (Table S6 and Figure S6).^23,41^ Methyl methacrylate (*m*/*z* 100), 2,4-dimethyl-1-heptene (*m*/*z* 126), 5-hexene-1,3,5-triyltribenzene (*m*/*z* 312), ε-caprolactam (*m*/*z* 113), 1,12-tridecadiene (*m*/*z* 180), and vinyl benzoate (*m*/*z* 148) were selected as indicator ions for PMMA,^23,24,39^ PP,^43^ PS,^23,39,44^ PA,^45,46^ PE,^47,48^ and PET,^41,47^ respectively. Specific indicator ions for these six polymers were not affected by tested natural materials (Table S8).^23,41^ Benzene (*m*/*z* 78) shows the highest peak intensity and sensitivity, while other components have much low sensitivity; thus, it was commonly selected as an indicator of PVC.^23,24^ However, natural materials and polymer PS and PET can interfere with benzene, so PVC was not considered in this study.

External calibration curves were obtained by analyzing different amounts of the standard plastics (0.1–10 μg for PMMA, PA, and PS and 0.1–200 μg for PP, PE, and PET) (Table S4). The identification of a single polymer in the sample was determined by comparison of the full-scan mass spectra of specific peaks with the analytical pyrolysis library (Figure S6).^26^ The instrument limits of detection and quantification (LOD and LOQ) were defined as 3 and 10 times the baseline noise, respectively (S/N = 3 and 10).^45^ LOD and LOQ values were then converted into procedural limits based on the volume of the original tested water samples (Table S5).

### Quality Assurance/Quality Control

Special care was required to minimize possible contamination during sampling, pretreatment, and detection procedures.^49^ In addition to the extraction and pretreatment of wastewater samples in an ordinary laboratory, the procedures including sample loading and drying were carried out in a fume hood. Cotton laboratory coats and polymer-free nitrile gloves (carefully washed before using) were used throughout the entire sampling and laboratory processes.^32,50,51^ Samples in containers were covered with aluminum foil to avoid potential airborne contamination. Stainless steel membranes, glass bottles, separation funnels, and the vacuum filtration device were rinsed three times with ultrapure water before use. During sampling and handing, several plastic materials such as ultrafiltration membranes, centrifugal tubes, and pipette tips were unavoidably used (Table S6), which were rinsed thoroughly three times with ultrapure water. The cross-flow ultrafiltration device was run for 10 min with ultrapure water to avoid possible interferences. All pyrolysis cups for Py-GC/MS were heated with a spirit lamp for at least 3 min before any samples were added to avoid any potential contamination.^39^

Three blank samples following the same steps as sample treatment, including microfiltration, ultrafiltration, digestion, drying, and sample loading, were prepared and detected with Py-GC/MS. As shown in Figure S4, there were significant peaks of benzene and styrene in the blank, but no specific compounds of the selected six polymers were identified in blank samples or the intensities were below detection limits, indicating that these treatment processes did not cause plastic contamination after careful cleaning. To determine the sample process efficiency, an extraction test was performed using PET, PS, and PP with high, medium, and low density. Three types of MPs with sizes of 100–400 and 1–50 μm and representative PS NPs with a size of 200 nm were added to the effluent wastewater and detected with the recovery experiment, which was performed according to the same procedure as the sampling and treatment.

## Results and Discussion

### Method Validation

The calibration range was from 0.1 to 10 μg for PMMA, PA, and PS and from 0.1 to 200 μg for PP, PE, and PET, where *R*^2^ ≥ 0.98 (Table S4). Each standard sample was repeated five times to determine the relative standard deviations (RSDs) of the quantitative ion peak areas, which were used to evaluate the precision of Py-GC/MS detection. The RSDs of the selected plastic polymers were 6.9–15.2% for PMMA, 4.2–19.0% for PP, 9.2–13.2% for PS, 3.9–16.9% for PE, 4.4–17.8% for PET, and 5.9–16.9% for PA. The recovery rates of selected MPs were 60.7–72.4, 64.8–75.4, and 65.5–79.1% for PP, PS, and PET, respectively (Table S7). Although the average recovery of MPs did not exceed 90%, it was acceptable and close to published values in other studies.^16,17,38^ The pretreatment processes including microfiltration, digestion, density separation, drying, and loading might result in certain sample loss of MPs. The major cause of loss would be physical adherence to container surfaces. Notably, the recovery of NPs (50.1–55.9%) was much lower compared with MPs in this study. First, 1 μm membrane filtration was likely to remove NPs that aggregated or absorbed on the surface of particles like MPs. Similar to MPs, the physical adherence to container surfaces was another main reason for low recovery. The concentration by cross-flow ultrafiltration also might cause large sample loss as suggested by Mintenig et al.^52^ Therefore, the proposed method caused a relatively large loss of NPs, but it was acceptable for the quantification of trace NPs in real wastewater, especially when there are no other mature quantitative methods.^35,42,52^

### Mass Concentration of Total MPs and NPs

**Figure 2:**
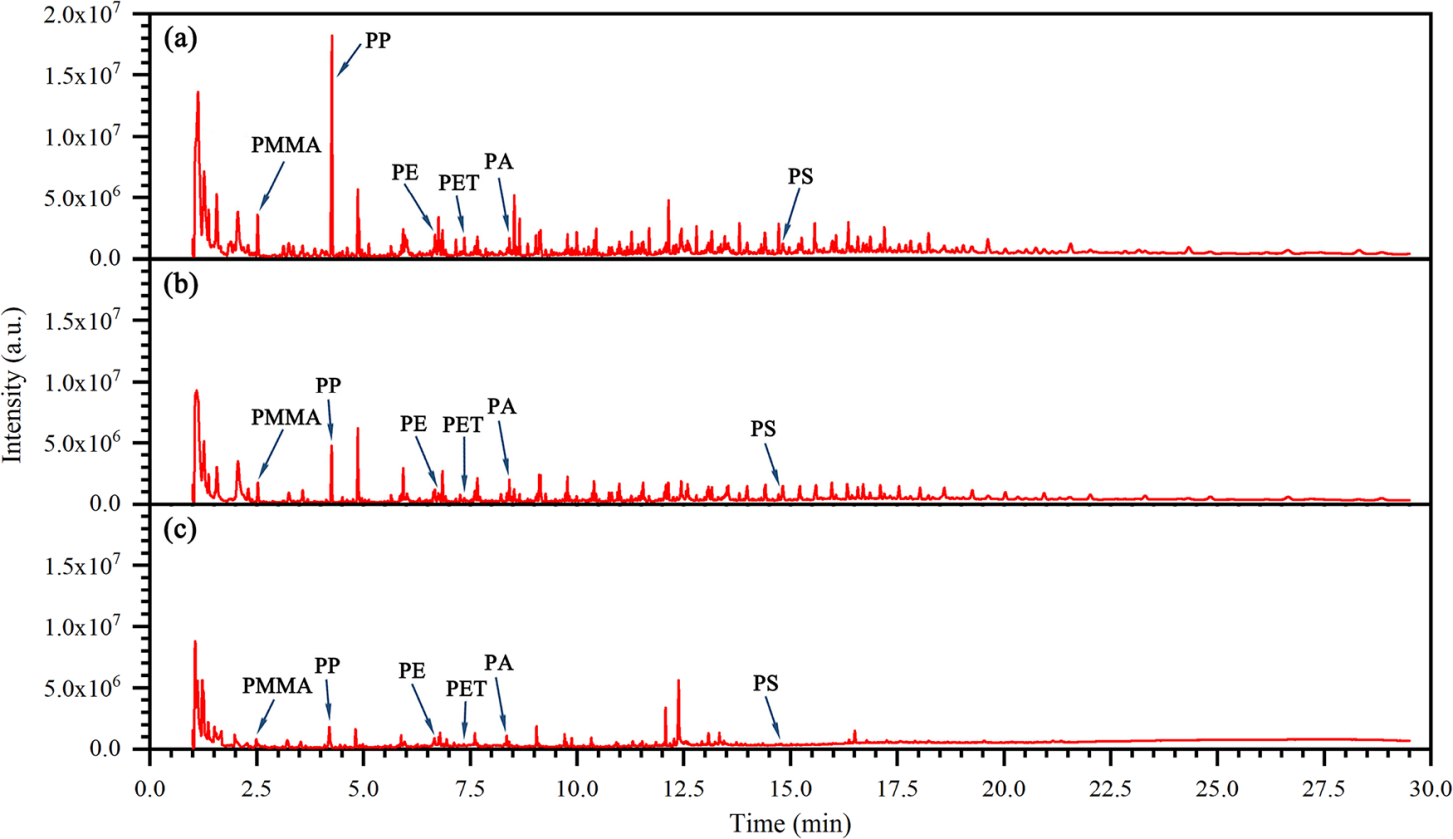
Chromatograms from representative samples of MPs with the size ranges of 50–1000 μm (a), 1–50 μm (b), and 0.01–1 μm (c).

Figure 2 shows the chromatograms of representative wastewater samples with sieving sizes of 50–1000, 1–50, and 0.01–1 μm. By analyzing the similarity analysis results of peaks (Figure S6), six specific peaks can be clearly distinguished at different retention times, which represented the characteristic ions of the selected plastic polymers. This suggested that Py-GC/MS was feasible for the identification of MPs, especially sub-MPs and NPs in complex wastewater samples. The mass concentration of MPs and NPs was quantified according to calibration curves of indicator ions and corresponding peak areas tested in samples. Table 1 shows the mass concentrations of total MPs and NPs at all sampling locations of both WWTPs. Generally, the total mass concentrations of MPs and NPs over plant A were calculated as 26.23 (influent), 9.50 (primary effluent), 7.62 (secondary effluent), and 1.75 μg/L (tertiary effluent) , and those of plant B were 11.29 (influent), 5.22 (primary effluent), 1.99 (secondary effluent), and 0.71 μg/L (tertiary effluent). Simon et al. reported that 2223–18,285 and 19–447 items/L MPs (10–500 μm) were found in influent and effluent wastewaters, respectively, and the mass concentrations were estimated as 61–1189 and 0.5–11.9 μg/L, respectively, according to particle size, density, and number.^17^ Quantified by TED-GC/MS, the mass concentration of MPs (>50 μm) in effluent wastewaters from a WWTP in Germany was 6.5–51.8 μg/L.^22^ The influent or effluent concentrations of MPs reported in these studies were relatively high compared to this study, which may be related to the pollution degree and treatment process of studied WWTPs as well as the differences in quantitative methods.^14^

**Table 1:** Mass Concentration (μg/L) and Removal Efficiency (%) of Total MPs in Plants A and B (*n* = 2)^a^

		sampling site			
WWTP	total MPs	0 (influent)	1 (primary effluent)	2 (secondary effluent)	3 (tertiary effluent)
plant A	mass concentration (μg/L)	26.23 ± 7.71	9.50 ± 1.22	7.62 ± 0.67	1.75 ± 0.02
	removal efficiency (%)	0	63.8	70.9	93.3
plant B	mass concentration (μg/L)	11.29 ± 0.71	5.22 ± 0.06	1.99 ± 0.52	0.71 ± 0.12
	removal efficiency (%)	0	53.8	82.4	93.7

a Locations 0, 1, 2, and 3 mean raw wastewater and treated water after primary, secondary, and tertiary treatment, respectively.

The mass concentration of MPs and NPs in WWTPs significantly decreased after undergoing whole treatment processes (Table 1), which corresponded with previous studies.^14,16^ MPs and NPs in plants A and B (63.8 and 53.8%, respectively) were removed by the primary treatment that was reported to remove 50–98% MPs in influents.^14^ This high removal efficiency was mainly attributed to the surface skimming and setting separation in primary clarifiers as well as the interception capability of the membrane screen with a pore size of 0.2–2 mm.^14^ The secondary treatment further removed 7.1 and 28.6% MPs and NPs in plants A and B, respectively, which were lower than the removal in the primary treatment. Although studies suggested that activated sludge flocs or extracellular polymeric substances and chemicals such as ferric sulfate or other flocculating agents used in biological tanks contributed to the accumulation and setting of MPs,^14,18^ the sludge return may result in the release of MPs, especially MPs with low density.^53^ Both of the studied WWTPs showed similar removal efficiency to MPs and NPs (93.3 and 93.7%, respectively) after the tertiary treatment, indicating that the tertiary treatment can effectively reduce the MP and NP pollution discharged from WWTPs into the aquatic environments.^28^ It was less effective compared with reported values (97–99.99%) of WWTPs with tertiary advanced treatment processes, such as the membrane bioreactor and rapid sand filtration in most studies.^16,18,29,54^ Unlike FTIR or Raman microspectroscopy that quantified MPs by counting, Py-GC/MS indirectly quantifying MPs and NPs by detecting the pyrolysis ions may produce certain errors, especially under low-weight loading.^55^ Moreover, the mass concentration of MPs and NPs in this study was not very suitable for comparison with a previous study on the particle numbers, that is, high particle number removal is essentially independent of high particle mass removal.^17^

### Polymer Type of MPs and NPs in Wastewater

**Figure 3:**
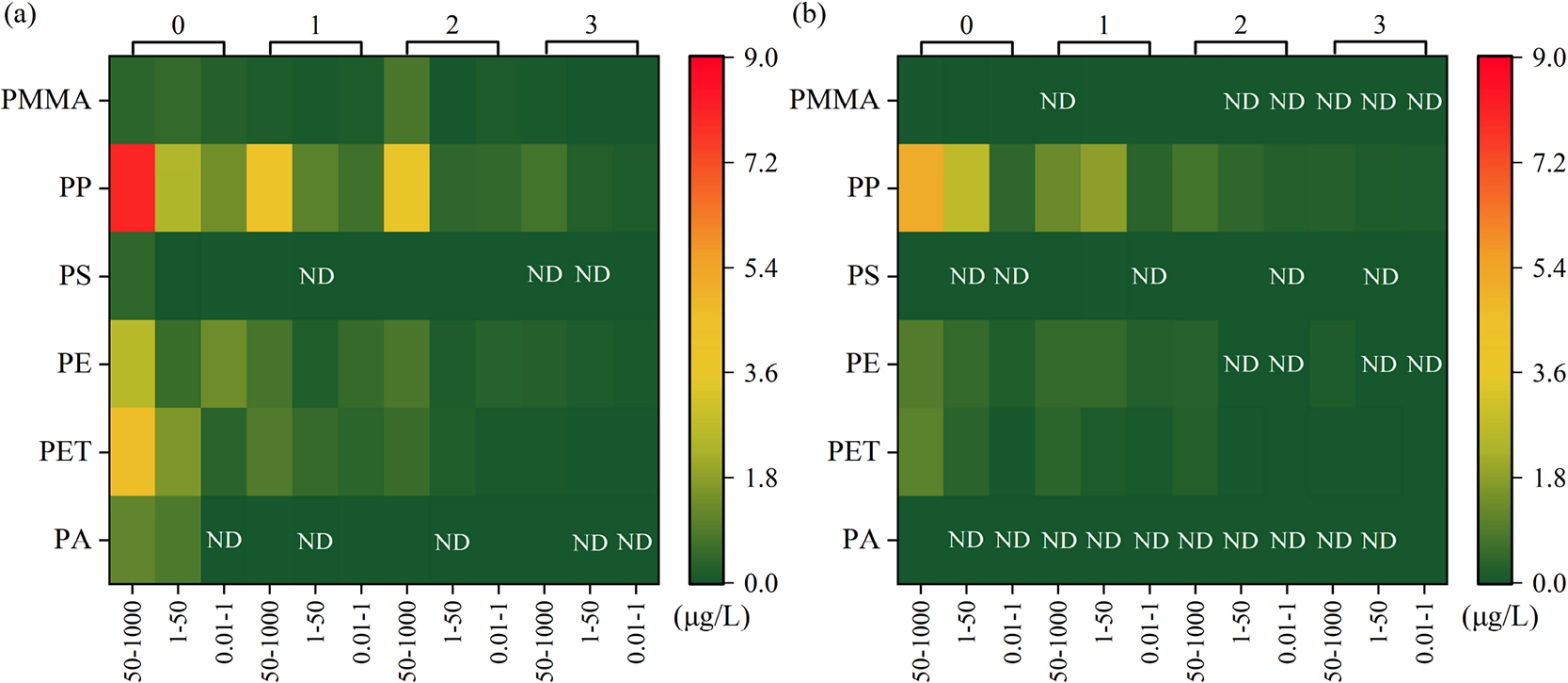
Heatmaps representing the average concentration of different types with different size ranges at the whole treatment processes of plants A (a) and B (b). Locations 0, 1, 2, and 3 mean raw wastewater and treated water after primary, secondary, and tertiary treatment, respectively.

**Figure 4:**
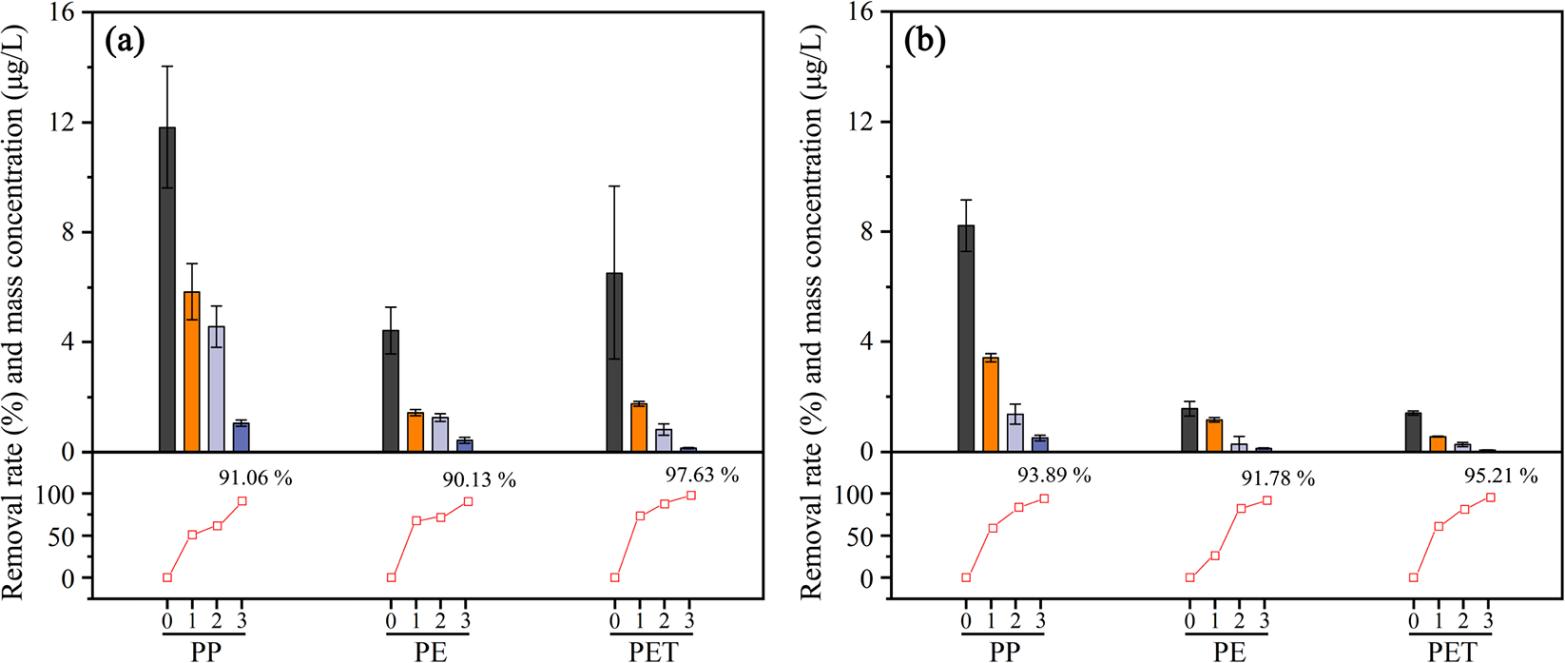
Mass concentration and removal efficiency of the main MPs and NPs (PP, PE, and PET) over the wastewater treatment process in plants A (a) and B (b). Locations 0, 1, 2, and 3 mean raw wastewater and treated water after primary, secondary, and tertiary treatment, respectively.

The heatmaps show the average mass concentrations of MPs and NPs with different size ranges at the whole treatment processes of two WWTPs (Figure 3). Almost all types of MPs and NPs with different size ranges except PS and PA were detected at all sampling sites in plant A (Figure 3a). However, several types of MPs and NPs were not detected or below the limit of detection at certain sites in plant B (Figure 3b). In plant A, PP, PET, and PE were the dominant material types in the influent, accounting for 45.1, 24.9, and 16.8%, respectively, with mass concentrations of 11.821, 6.524, and 4.412 μg/L followed by PA (1.977 μg/L, 7.5%), PMMA (1.052 μg/L, 4.0%), and PS (0.445 μg/L, 1.7%). However, in plant B, the proportion of PP in the influent was the largest (8.219 μg/L, 72.8%) followed by PE (1.564 μg/L, 13.9%), PET (1.408 μg/L, 12.5%), PMMA (0.089 μg/L, 0.8%), PA (0.009 μg/L, 0.08%), and PS (0.003 μg/L, 0.03%). Considering the low concentration of PMMA, PS, and PA, the following discussion is mainly focused on PP, PE, and PET (Figure 4). The abundance of different types of MPs and NPs differed somewhat in both plants, which might be related to the production, export, and usage of relevant plastic products in the two regions discharging wastewater to the plants. As reported, PET (60%) and PE (14%) were prevalent types of MPs in Finnish WWTP influents.^28^ The percentages of PP, PE, and PET were 39.6, 25.6, and 21.3%, respectively, in Korea’s WWTP influents.^16^ Similarly, PP, PE, PS, and PET accounting for 30.2, 26.9, 10.3, and 7.5%, respectively, were found in other WWTP influents in China.^19^ Although the proportion of MPs and NPs in this study was evaluated by the mass concentration rather than particle number, PP, PE, and PET appeared to be the most popular polymers in the influent of most WWTPs. In the effluents, PP, PE, and PET were also the dominant polymers, with average mass concentrations of 1.057, 0.436, and 0.155 μg/L in plant A and 0.503, 0.129, and 0.068 μg/L in plant B, respectively (Figure 4). Several studies also quantified MPs in the effluent of WWTPs based on thermal analytical methods. Goedecke et al. reported that 1.89–46.42 μg/L PE, 0–35.35 μg/L PP, and 1.60–8.14 μg/L PS MPs were found in the effluent of a German WWTP.^22^ Only PE (81–257 μg/L) and PS MPs (0.072 μg/L) were detected in the effluent of two other German WWTPs by Majewsky et al.^56^ and Funck et al.,^57^ respectively. The effluent MP type and mass reported in German WWTPs were relatively high compared with the studied WWTPs here, which may be related to the differences in sampling and analysis methods, MP pollution sources, and wastewater treatment processes.

The proportion of different types of MPs and NPs in raw wastewater changed after a series of treatment processes. In plant A, the proportion of PP and PE increased from 45.1 and 16.8 to 60.3 and 24.8%, but the proportion of PET decreased from 24.9 to 8.8% in plant A after tertiary treatment. In plant B, the proportion of PE in the effluent was higher than in the influent, while the proportion of PET was lower than that in the influent. This may be attributed to the different removal efficiencies of WWTPs toward different types of MPs and NPs.^16,19^ As calculated, the removal efficiencies of PP and PE in plant A were 91.1 and 90.1%, respectively, lower than that of PET (97.6%) (Figure 4a). Similarly, the higher percentage of PET was removed in plant B compared with PP and PE (Figure 4b). Long et al. also reported that the removal efficiencies of PP, PE, and PET were 92.0, 87.8, and 96.4%, respectively.^19^ The polymer density might be the main reason for this. Compared with PET, PP and PE with relatively low density tended to float on the surface of wastewater and relatively few particles were captured by suspended solids or activated sludge and eventually removed.^19^ Moreover, the settled MPs, especially the low-density ones, may be partly resuspended and released into wastewater under turbulent mixing.^19,58^ Another explanation was that PET was usually in the shape of elongated fibers, which may make it easy to entangle and remove during wastewater treatment.^16,28^

### Size Distribution of MPs and NPs

**Figure 5:**
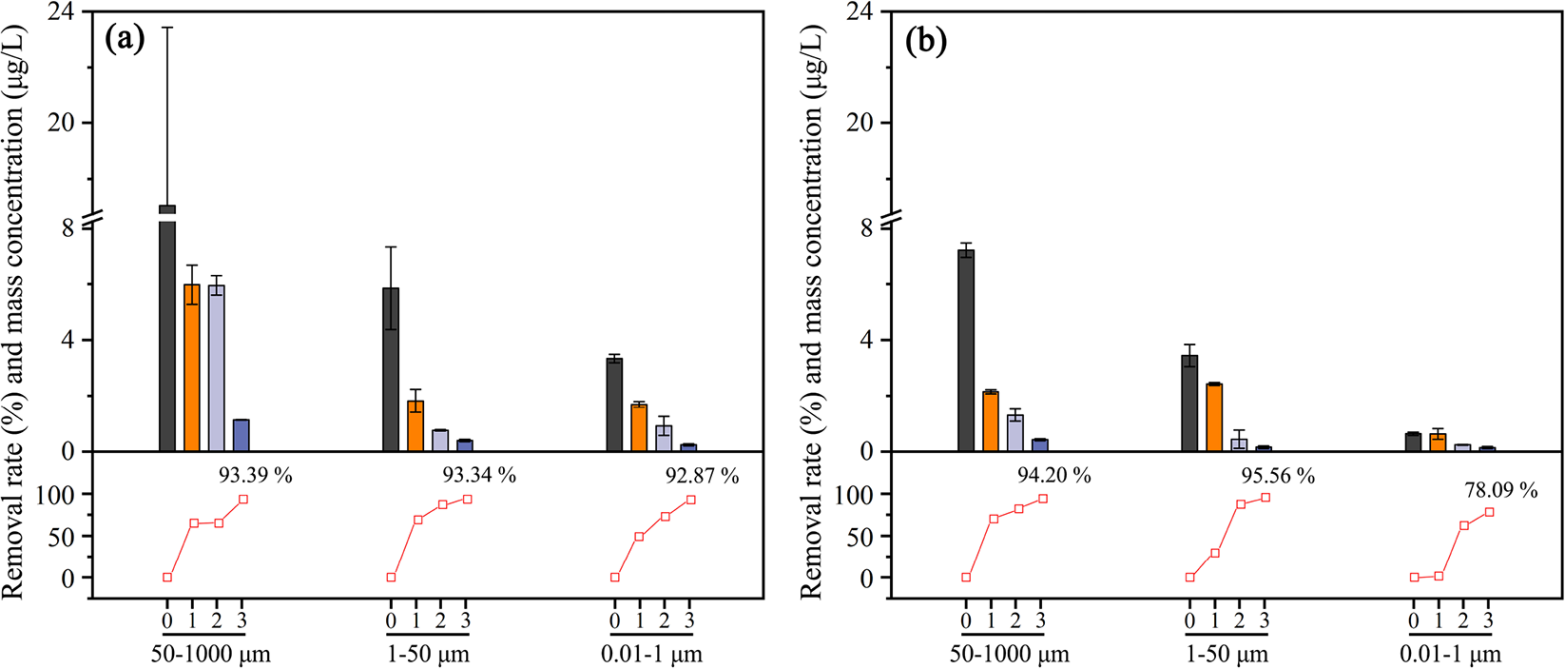
Mass concentration and removal efficiency of MPs with different size ranges in the whole wastewater treatment process in plants A (a) and B (b). Locations 0, 1, 2, and 3 mean raw wastewater and treated water after primary, secondary, and tertiary treatment, respectively.

Figure 5 shows the mass concentrations of MPs and NPs with different size ranges in wastewater of both plants. In plant A, MPs with a large particle size (50–1000 μm) accounted for 62.9–78.1% from the influent to the effluent, higher than MPs and NPs of 1–50 μm (10.0–22.3%) and NPs of 0.01–1 μm (12.0–17.9%) (Figure 5a). Similarly, MPs of the three particle size ranges in wastewater from plant B are 41.3–65.7, 21.5–46.7, and 5.6–19.5% (Figure 5b). Generally, the proportion of MPs and NPs decreased with the decreasing particle size, which was contrary to the results of several studies.^51,59,60^ Jiang et al. reported that the proportions of the 20–100 μm (34.0–49.7%) and 100–500 μm (29.0–48.5%) MPs were higher than MPs with the larger size in all wastewater samples.^60^ Pivokonsky et al. also found that MPs of 1–5 and 5–10 μm accounted for 40–60 and 30–40% of the total MPs, respectively.^59^ In this study, the concentration of MPs was quantified by mass rather than the particle number as investigated in previous studies. When the particle size was small enough, the mass can be very low, even though the particle number was very high. Therefore, previous quantitative methods may result in an overestimation of the concentration of MPs and NPs with small size because MPs and NPs were likely to be fragmented after physical and chemical processes in WWTPs.^21^ The mass investigated in this study is important to evaluate the relationship between the quantity and size of MPs, especially when there is no suitable method to quantify sub-MPs and NPs in WWTPs. It should be noted that part of NPs in wastewater that aggregated or adsorbed onto MPs and other particles was filtered out by the membrane filtration step. The exclusion of those NPs may slightly underestimate the proportion of NPs in the actual wastewater samples.

Figure 5 also shows the removal efficiency of MPs with different size ranges. In plant A, the total removal efficiency of MPs and NPs with a size of 50–1000 μm was 93.39%, higher than those of 1–50 μm (93.34%) and 0.01–1 μm (92.87%) (Figure 5a). The lowest removal efficiency of MPs with a size of 0.01–1 μm (78.09%) compared with other size ranges of MPs was also found in plant B (Figure 5b). Although the total removal efficiency seemed to decrease with the decrease in particle size, different treatment processes had different removal efficiencies for MPs and NPs with different size ranges. Conventional primary treatment including the screen and sedimentation tank that were designed to remove large suspended particles was more efficient in removing MPs with larger size.^14,29^ MPs and NPs with smaller sizes seemed to be removed more easily by the activated sludge tank. The density affected the vertical distribution of larger particles, but Brownian motion determined the fate of smaller particles.^61,62^ Therefore, large MPs with low density may get resuspended under turbulent mixing while the hetero-aggregation between small particles and activated sludge contributed to their removal in the activated sludge tank.^19,58^ Studies also suggested that the short residence time of smaller-sized MPs caused by the fast fragmentation and degradation was another reason for higher removal efficiency in secondary treatment.^19^ The tertiary advanced treatment was necessary to reduce MP and NP pollution in the effluent but was not effective in removing small MPs and NPs, which was probably due to the fact that these small particles could pass the sand filter or membrane filtration more easily.^14^

### Input and Output of MPs and NPs in WWTPs

The annual input and output of MPs and NPs in WWTPs were estimated based on the total amount of wastewater of both plants (1.83 × 10^8^ and 0.73 × 10^8^ m^3^/year for plants A and B, respectively). Plants A and B received approximately 4.801 and 0.824 tons of MPs and NPs, respectively, in 1 year. The amounts of MPs and NPs released into the aquatic environment were 0.321 and 0.052 tons per year for plants A and B, respectively. Simon et al. also estimated slightly more than 3 tons/year of MP discharge from WWTPs in Denmark, indicating that WWTPs contributed little to MP emissions.^17^ The emissions of NPs could be negligible, with annual emissions from plants A and B being 0.044 and 0.010 tons, respectively. However, low NP emissions do not mean they do not need a lot of attention. Photodegradation of MPs may occur when MPs are discharged into the aquatic environment, which may convert MPs to NPs and increase NP levels.^63,64^ In addition, NPs may pose a higher ecological risk than MPs as these particles can be easily absorbed by organic organisms when they reach the nanometer scale.^9,65−68^ Therefore, this highlights the need for tertiary treatment technologies to remove small MPs, especially NPs from the effluent.

## Environmental Implications

The mass concentrations of MPs and NPs in WWTPs were quantified by Py-GC/MS in this study. The total mass concentrations of MPs in two WWTPs, plants A and B, decreased from 26.23 and 11.28 μg/L in the influent to 1.75 and 0.71 μg/L in the effluent, respectively. Among them, PP, PET, and PE were the dominant polymer types in wastewater, while PMMA, PS, and PA only accounted for a small part. The mass concentrations of NPs (<1 μm) were much lower than those of MPs (>1 μm), accounting for 12.0–17.9 and 5.6–19.5% of the total MPs and NPs, respectively. In total, 93.3 and 93.7% of MPs and NPs were removed in plants A and B, respectively. Notably, the removal efficiency differed with the polymer type and size range. The low-density MPs (e.g., PP and PE) had lower removal efficiency than high-density PET in both plants. Since MPs and NPs with smaller particle size could pass the tertiary sand filter or membrane filter more easily, the removal efficiency of NPs was lower than that of MPs with larger particle size. Overall, in this study, the mass concentration of MPs and NPs with particle sizes ranging from 0.01 to 1000 μm in wastewater was studied by Py-GC/MS, providing new insights into the pollution level and removal characteristics of MPs and NPs in WWTPs. Since it is less affected by differences in handing procedures and target size ranges, the mass concentration is a more robust measurement.

It is important to notice that there are limitations and uncertainties in the current study, especially regarding the sample collection and MP/NP detection. For example, the practical limitations of field wastewater sampling may influence the calculated MP inputs and outputs, such as the daily variations in weather, flow, and pollutant concentrations.^14,18^ In addition, the used plastic items (e.g., sampling buckets, UF membranes, centrifugal tubes, and pipette tips) may introduce potential contamination. Though no contamination was detected in the blank samples, the usage of plastic items should be avoided as much as possible in future studies. Moreover, only several individual plastic polymers with relatively high abundance in WWTPs were studied, while it is not easy to detect unusual plastic polymers or plastic composites by Py-GC/MS.^19^ Thus, some plastic composites (e.g., PE-PP) and polymers with similar structures (e.g., different kinds of polyester) may interfere with the detection of the selected plastic polymers. Additionally, the proposed method caused loss of MPs, especially NPs, which can lead to underestimation of MPs/NPs. Further studies are recommended to address these challenges about MPs/NPs in wastewater.

## Supporting Information Available

Determination of standard curves of selected plastics (Text S1); cross-flow ultrafiltration (Text S2); selection of indicator ions of different plastic polymers (Text S3); extraction and recovery efficiency of MPs and NPs (Text S4); SEM images of raw plastics, methanol, 65 °C treated plastics, and 90 °C treated plastics (Figure S1); total ion chromatogram pyrograms and the mass spectra of the characteristic pyrolysis products of six selected plastics (Figure S2); chromatograms of selected natural polymers (Figure S3); chromatogram of a representative blank sample (Figure S4); chromatograms of representative samples with different size ranges (Figure S5); results of similarity analysis in the characteristic peaks of a representative sample (Figure S6); effect of 90 °C heating on the mass of selected plastics (Table S1); polymer density and ratio of methanol/dichloromethane (Table S2); conditions for single-shot Py-GC/MS measurements (Table S3); characteristic components and calibration functions of six plastics (Table S4); LOD and LOQ of different plastics at different sampling sites (Table S5); the composition of plastic materials used in sampling and sample preparation (Table S6); recovery of selected MPs and NPs (Table S7); potential interferences of selected natural polymers (20 mg) with polymer indicator ions (Table S8); mass concentrations of PMMA, PP, PS, PE, PET, and PA measured in two WWTPs (Table S9) (PDF)
