# Supplementary material for: Assessing the Mass Concentration of Microplastics and Nanoplastics in Wastewater Treatment Plants by Pyrolysis Gas Chromatography–Mass Spectrometry
Source: Environ Sci Technol. 2023 Feb 14;57(8):3114–23. doi: 10.1021/acs.est.2c07810 (PMC9979646; doi:10.1021/acs.est.2c07810)
Supplement: Supplementary file 1 — es2c07810_si_001.pdf [file es2c07810_si_001.pdf]

Supplementary Information for:

Assessing Mass Concentration of Microplastics and Nanoplastics in Wastewater Treatment Plants by Pyrolysis Gas Chromatography-Mass Spectrometry

Yanghui Xu [a, b], Qin Ou [a, b], Xintu Wang [a, c], Feng Hou [d], Peng Li [d], Jan Peter van der Hoek [b, e], Gang Liu [a, b, f], *

[a] *Key Laboratory of Drinking Water Science and Technology, Research Centre for Eco-Environmental Sciences, Chinese Academy of Sciences, Beijing, 100085, P. R. China*

[b] *Section of Sanitary Engineering, Department of Water Management, Faculty of Civil Engineering and Geosciences, Delft University of Technology, Stevinweg 1, 2628 CN Delft, the Netherlands*

[c] *College of Environmental Science and Engineering, Guilin University of Technology, Guilin, Guangxi province, 541004, R.R. China*

[d] *China Water Environmental Group Limited, Jinbao Street 89, 101101, Beijing, P.R. China*

[e] *Waternet, Department Research & Innovation, P.O. Box 94370, 1090 GJ Amsterdam, the Netherlands*

[f] *University of Chinese Academy of Sciences, Beijing, 101408, P.R. China*

*Corresponding authors：

Prof. Dr. Gang Liu,
Research Center for Eco-Environmental Sciences,
Chinese Academy of Sciences,
Beijing, China
email: gliu@rcees.ac.cn
Tel: 008617600879707

The supplementary information includes 4 texts, 6 figures, and 9 tables in 21 Pages.

Text S1. Determination of standard curves of selected plastics.

Direct weighting of certain quantities of the polymers was impossible because of uncertainties of the weighting of small quantities when preparing the lower calibration levels. The preparation of different calibration concentrations from a stock solution by serial dilution was conducted. Due to the poor solubility of several polymers like PE and PP in the most common solvents, the mixture of dichloromethane and methanol was used to disperse seven plastic polymers. Different plastic polymers have different densities, thus dichloromethane and methanol were mixed in certain proportions yielding a solution with a density close to that of these polymers (Table S2). 100 mg polymer powder was weighed and dissolved in 10 mL mixed organic solvent to obtain a plastic dispersion with a concentration of 10 g/L. The stock solution was continuously diluted to obtain 2−1000 mg/L (2, 4, 20, 40, 200, 400, and 1000 mg/L) plastic dispersion. The 50 μL standard plastic solution was transferred to an 80 μL pyrolysis cup and dried at 60 °C in a drying oven to obtain different calibration concentrations of different plastic polymers (Table S4).

The calibration curves of seven plastic standard polymers are positive and linear in the range of 0.1−10 μg (0.1, 0.2, 1, 2, and 10 μg) for PMMA, PA, PS, PP, PE and PET, and 10−200 μg (10, 20, 50, 100, and 200 μg) for PP, PE and PET with acceptable determination coefficients ($R^2 \geq 0.98$) (Table S4). The relative standard deviations (RSDs) of the quantitative ion peak area with 5 replicates for each standard sample were used to evaluate the precision of this method measured by Py-GC/MS.

Text S2. Cross-flow Ultrafiltration.

The collected samples were filtered with 50 μm and 1 μm stainless steel meshes on a glass filtration unit. Next, the filtrate collected in a water tank was injected into a cross-flow ultrafiltration system with a molecular weight cutoff of 100 KDa. The clean water was discharged and the wastewater was injected into the tank again to achieve the concentration of raw water. After approximately 2 h, water sample was filtered and concentrated into a volume of approximately 200 mL. The final retentate was concentrated approximately 250 times. Additionally, cross-flow ultrafiltration can remove most of the dissolved organic matter because the molecular weight of dissolved organic matter in the aquatic environment is almost below 100 KDa.[1-4] In order to avoid background contamination and cross-contamination, the ultrafiltration cartridge was injected with ultrapure water and cleaned for 10 min prior to water sample concentration, and replaced when concentrating another water sample.

Text S3. Selection of indicator ions of different plastic polymers

As benzene (m/z 78) shows the highest peak intensity and sensitivity while other components have much low sensitivity, benzene was selected as an indicator of PVC.[5, 6] For PMMA, methyl methacrylate (m/z 100) was selected as an indicator compound which is specific and high-sensitivity.[5-7] 2, 4-Dimethyl-1-heptene (m/z 126) was specific and considered as an indicator ion for PP,[8] and the molecular ion m/z 43 with a high response was selected as the quantification ion. For PS, styrene monomer shows a high peak intensity but it may be produced from environmental matrixes like chitin and albumin, so the specific styrene trimer (5-hexene1, 3, 5-triyltribenzene, m/z 312) was considered as an indicator compound and the high-response molecular ion m/z 91 was selected as the quantification ion of PS.[5, 7, 9] 1-decene was selected as the indicator ion for PE as it is the most representative pyrolysis component with high sensitivity.[6, 10] For PET, benzoic acid (m/z 105) with high peak intensity and sensitivity was selected as an indicator component.[5, 10, 11] ε-caprolactam (m/z 113) is a specific pyrolysis product with high sensitivity, so it was considered as an indicator ion for PA.[12, 13]

Quantification of MPs and NPs by Py-GC/MS is based on the indirect determination by analyzing their pyrolysis products, but these products also be produced from natural matters present in water samples. The selectivity of the indicator compounds was tested by analyzing several selected organic materials including wood, leaf, fish, humic acid,

and black carbon (Table S6).[5, 10] The indicator ions for PMMA, PP, PE, PS, PET and PA were not affected by these natural materials.[5, 10] It cannot confirm that the pretreatment can completely remove the interference for PVC because benzene was abundant in most natural matters.[5, 6, 10] Thus the quantification of PVC in the water samples was potentially subject to minor bias from natural materials.

Text S4. Extraction and recovery efficiency of MPs and NPs.

In order to determine the sample process efficiency, an extraction test was performed using PET, PS and PP with high, medium and low density. Three material types of MPs with size of 100–400 μm and 1–50 μm, and representative PS NPs with size of 200 nm were added the effluent wastewater (25 L) and detected with the recovery experiment was performed according to the same procedure as the pretreatment including microfiltration, ultrafiltration, digestion, density separation, drying and loading. Three samples were simultaneously spiked 20, 10, 5 μg/L MPs with size of 100–400 μm and 1–50 μm, and PS NPs to test the extraction and recovery efficiency of MPs. Recovery rates were calculated using the following equation:

$$\text{recovery (\%)} = \frac{C_2 - C_0}{C_1} \times 100 \qquad \text{(S1)}$$

where C_0 (μg/L) is mass concentration of MPs in the control samples without spiked and C_2 (μg/L) is mass concentration of MPs detected in samples spiked with a known concentration C_1 (μg/L).

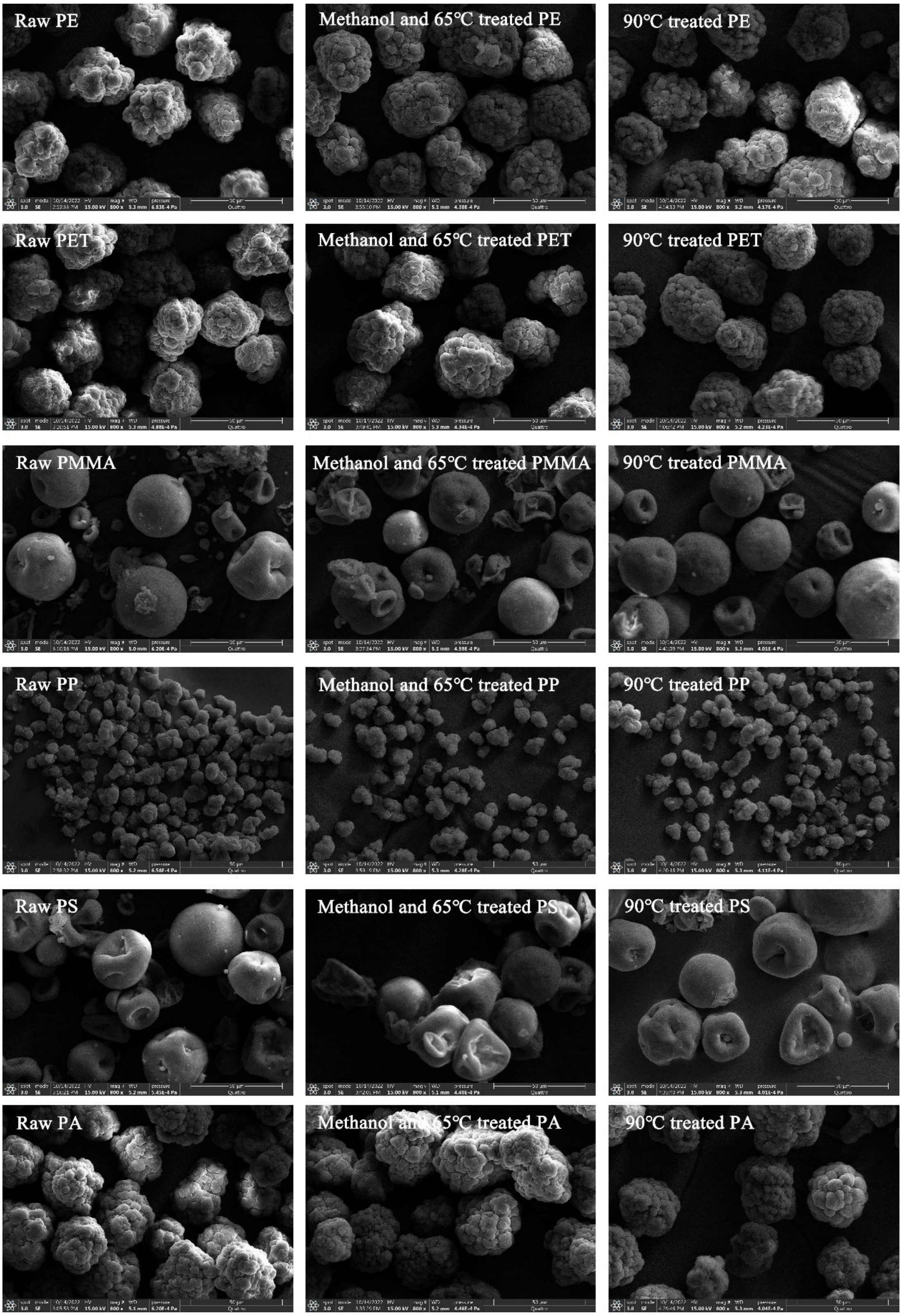

Figure S1. SEM images of raw plastics, methanol and 65°C treated plastics and 90°C treated plastics.

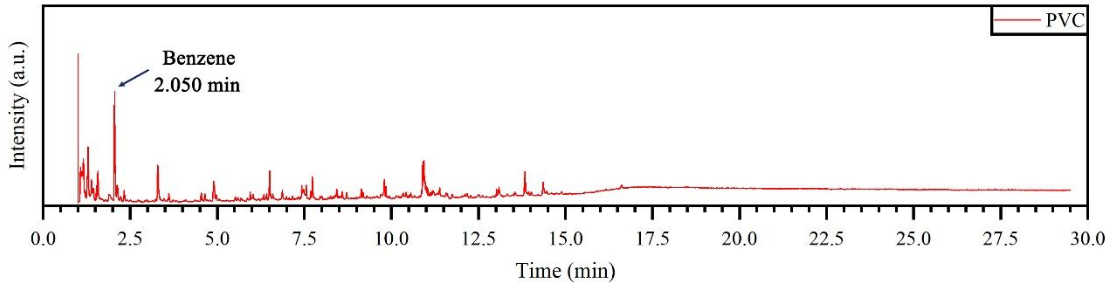
PVC
Benzene
2.050 min
Intensity (a.u.)
0.0 2.5 5.0 7.5 10.0 12.5 15.0 17.5 20.0 22.5 25.0 27.5 30.0
Time (min)

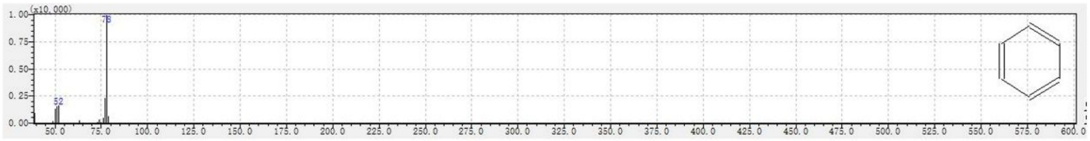
(x10,000)
78
52

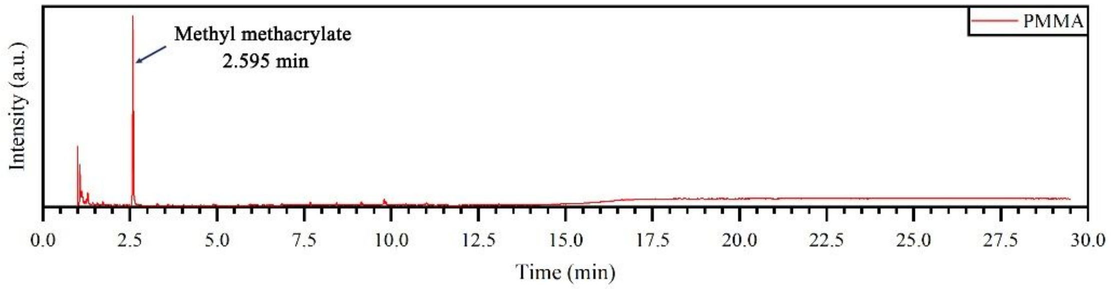
PMMA
Methyl methacrylate
2.595 min
Intensity (a.u.)
0.0 2.5 5.0 7.5 10.0 12.5 15.0 17.5 20.0 22.5 25.0 27.5 30.0
Time (min)

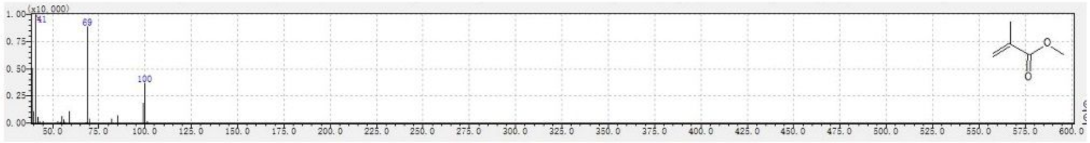
(x10,000)
41
69
100

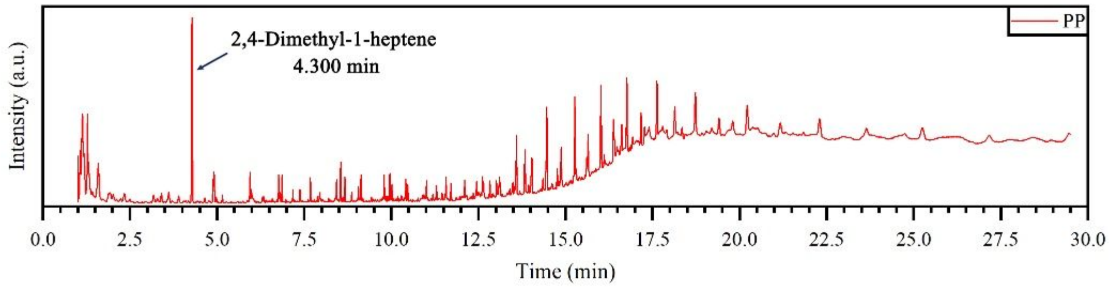
PP
2,4-Dimethyl-1-heptene
4.300 min
Intensity (a.u.)
0.0 2.5 5.0 7.5 10.0 12.5 15.0 17.5 20.0 22.5 25.0 27.5 30.0
Time (min)

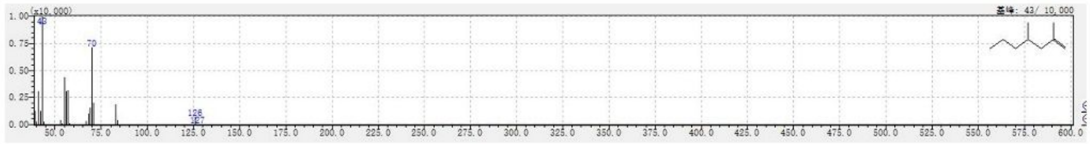
(x10,000)
70

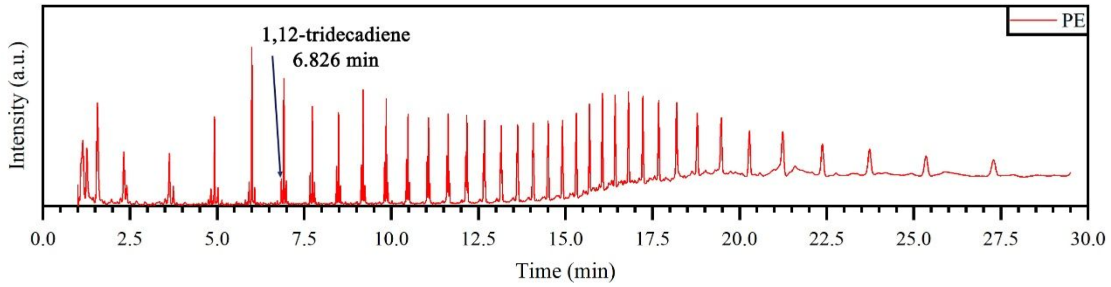
PE
1,12-tridecadiene
6.826 min
Intensity (a.u.)
Time (min)

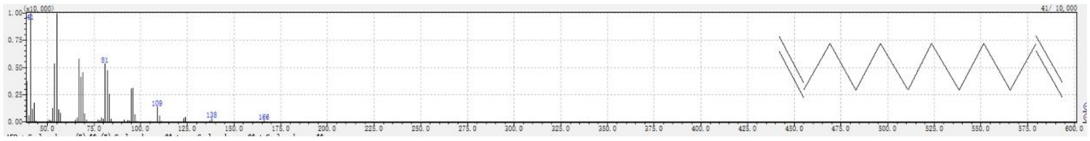

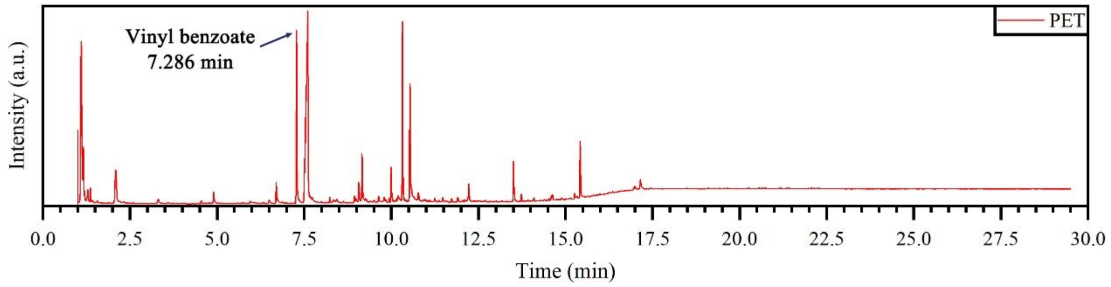
PET
Vinyl benzoate
7.286 min
Intensity (a.u.)
Time (min)

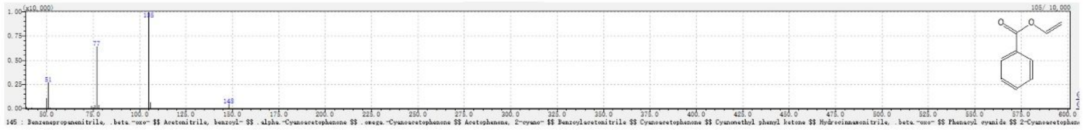

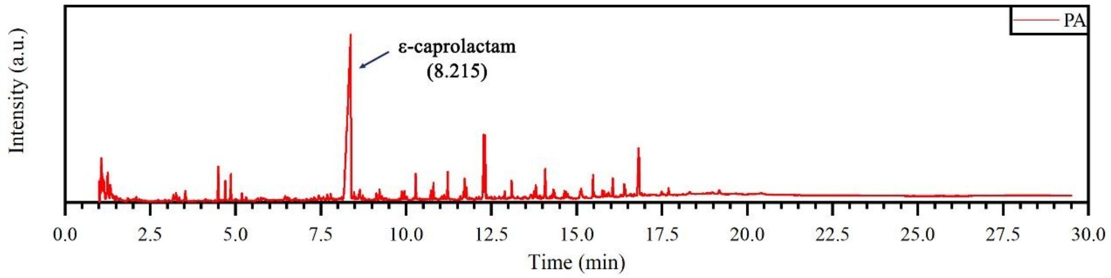
PA
ε-caprolactam
(8.215)
Intensity (a.u.)
Time (min)

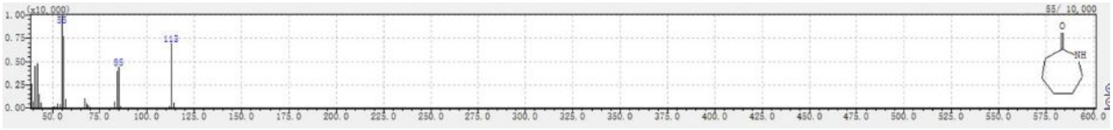

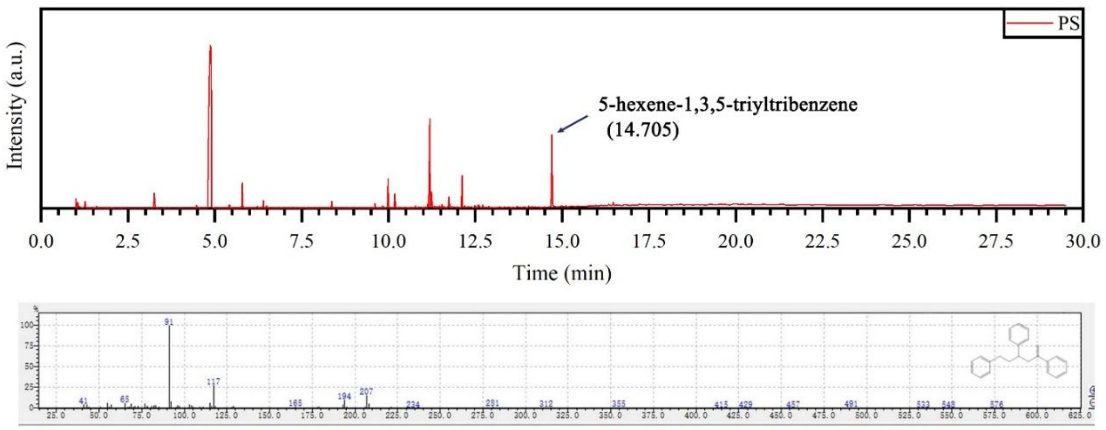

Figure S2. Total ion chromatogram pyrograms and the mass spectra of the characteristic pyrolysis products of six selected plastics.

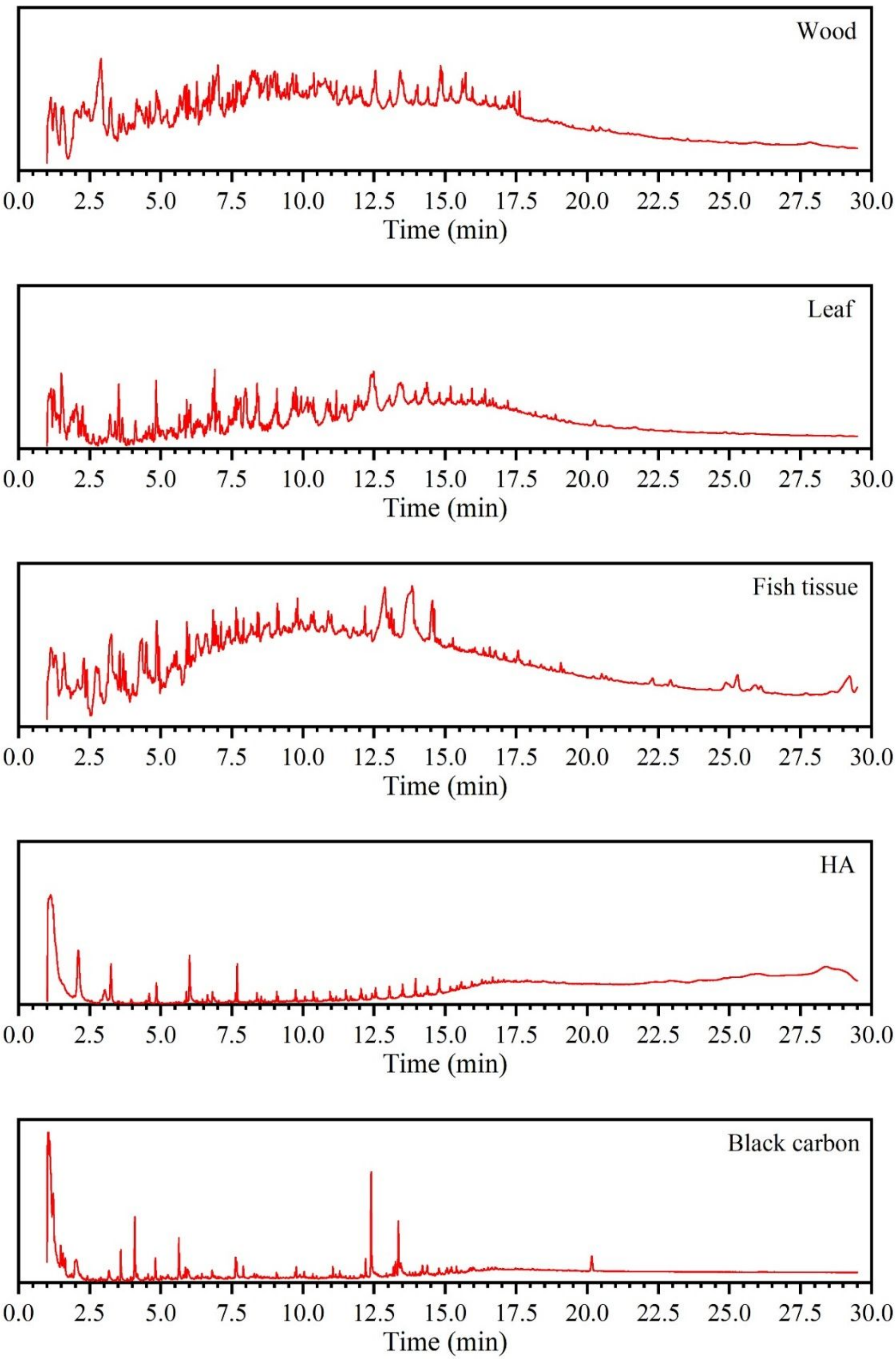

Figure S3. Chromatograms of selected natural polymers.

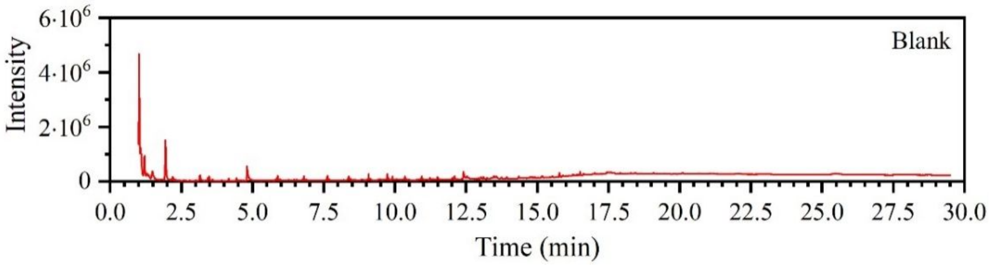

Figure S4. Chromatogram of a representative blank sample.

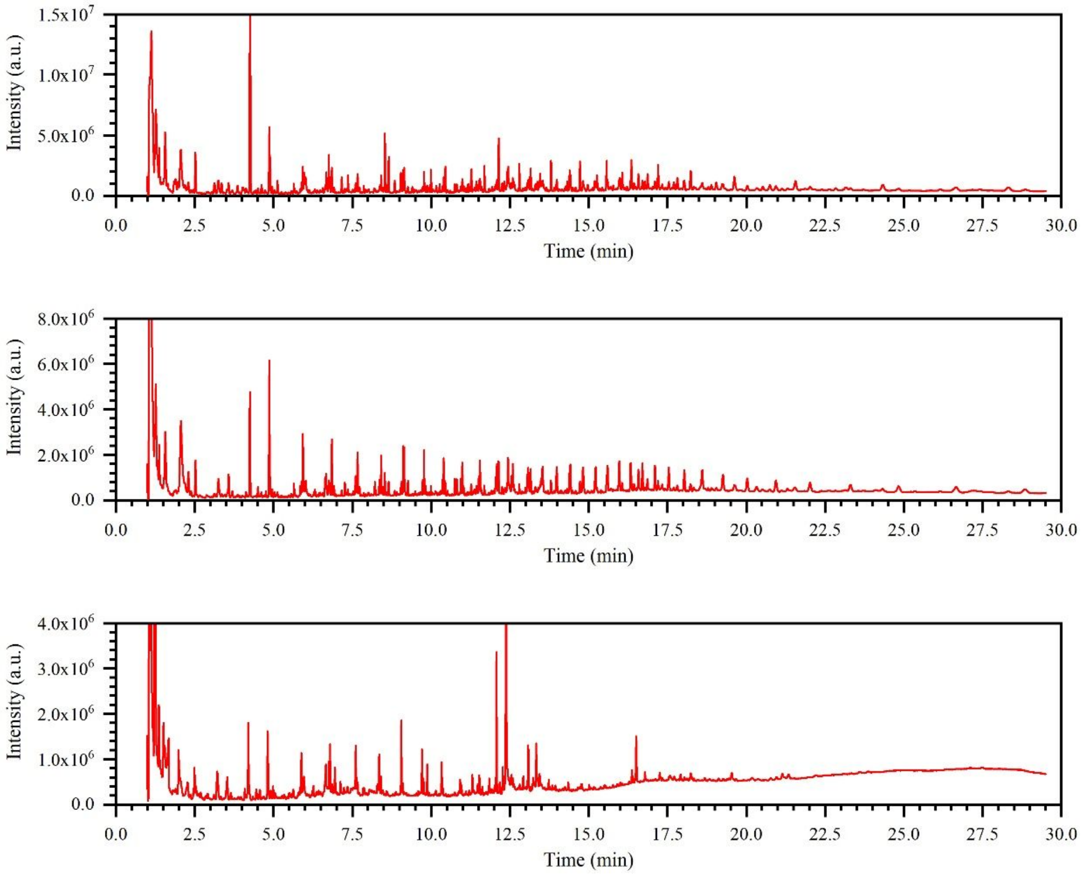

Figure S5. Chromatograms of representative samples with different size range.

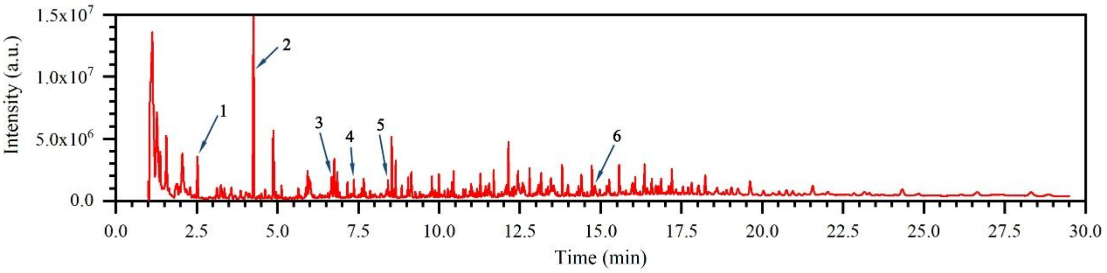
Intensity (a.u.)
Time (min)
1
2
3
4
5
6

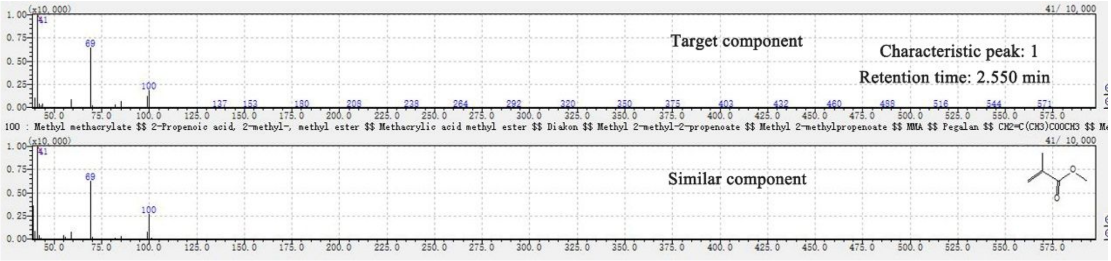
Target component
Characteristic peak: 1
Retention time: 2.550 min
100 : Methyl methacrylate $$ 2-Propenoic acid, 2-methyl-, methyl ester $$ Methacrylic acid methyl ester $$ Diakon $$ Methyl 2-methyl-2-propenoate $$ Methyl 2-methylpropenoate $$ MMA $$ Pegalan $$ CH2=C(CH3)COOCH3 $$
Similar component

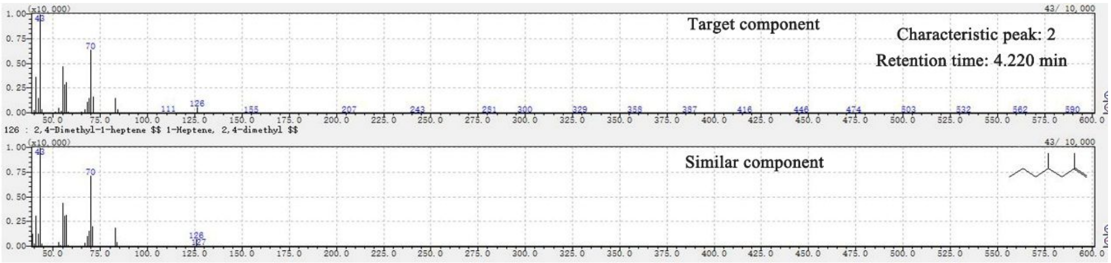
Target component
Characteristic peak: 2
Retention time: 4.220 min
126 : 2,4-Dimethyl-1-heptene $$ 1-Heptene, 2,4-dimethyl $$
Similar component

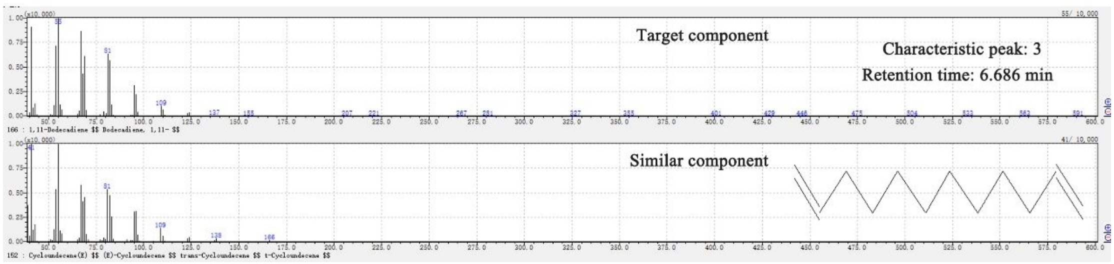
Target component
Characteristic peak: 3
Retention time: 6.686 min
Similar component

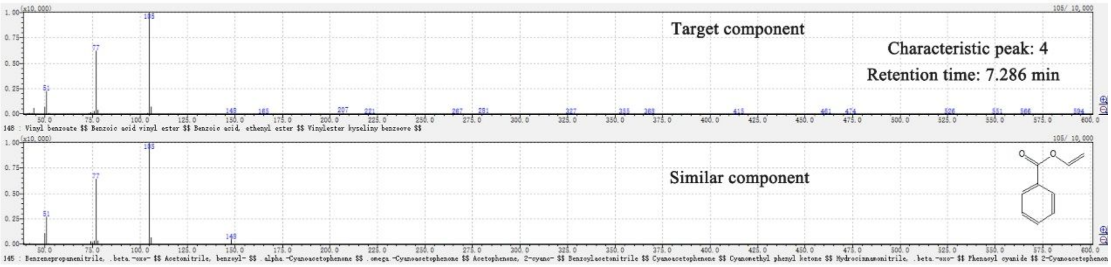
Target component
Characteristic peak: 4
Retention time: 7.286 min
Similar component

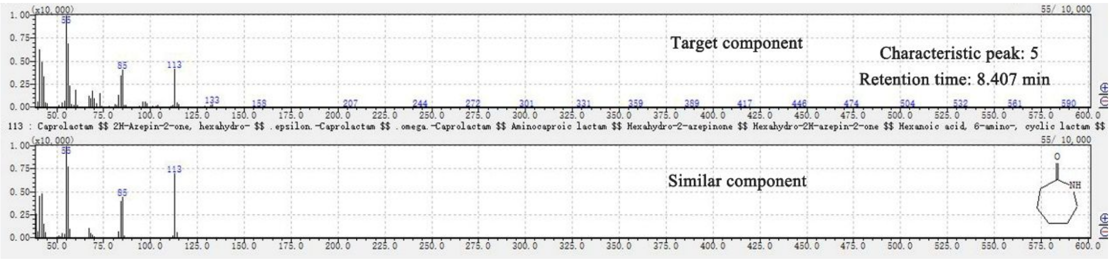

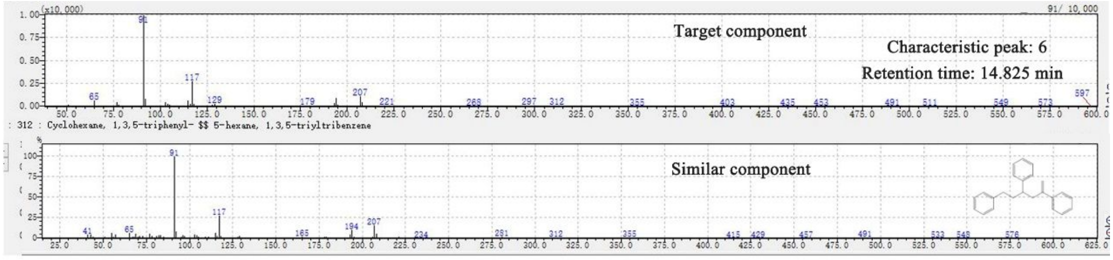

Figure S6. Results of similarity analysis the characteristic peaks of a representative sample.

Table S1. Effect of 90 °C heating for 12 h on the mass of selected plastics.

MPs	m_0 (mg)	m_1 (mg)	m_0 (mg)	m_1 (mg)	Recovery (%)
PMMA	20.20	20.17	20.14	20.10	99.83 ± 0.02
PP	20.03	20.02	20.22	20.12	99.73 ± 0.22
PE	20.17	20.16	20.65	20.67	100.27 ± 0.17
PS	20.21	20.22	20.08	20.07	100.00 ± 0.05
PET	20.08	20.07	20.07	20.07	99.98 ± 0.02
PA	20.03	19.98	20.24	20.24	99.88 ± 0.12

Table S2. Polymer density and ratio of methanol/dichloromethane.

Polymer type	Density (g/cm^3)	Methanol/dichloromethane
PMMA	1.19	0.34
PP	0.89	4.41
PS	1.03	1.23
PE	0.914	3.35
PET	1.08	0.85
PA	1.25	0.16

Table S3. Conditions for single shot Py-GC/MS measurements.

**Micro-furnace pyrolyzer**	
Carrier gas	helium
Pyrolysis time	0.2 min
Pyrolysis temperature	650 °C
Interface temperature	320 °C
**Gas chromatograph**	
Column	DB5 (J&W); 30 m, 0.25 mm I.D., 0.25 μm film thickness
Injector port temperature	320 °C
Temperature program	40 °C (2 min) → 320 °C (20 °C/min, 14 min)
Mode	50:1
**Mass spectrometer**	
Ionization energy	70 eV
Scan range	10-600 *m/z*
Ion source temperature	250 °C

Table S4. Characteristic components and calibration functions of six plastics.

Plastic type	Characteristic components	Linear range	Calibration functions	Linearity (R^2)	RSD (%)
PMMA	Methyl methacrylate	0.1−10 μg	y = 413336x - 42490	0.99	6.9−15.2
PP	2, 4-Dimethyl-1-heptene	0.1−10 μg	y = 112249x + 15378	0.99	11.3−19.0
		10−200 μg	y = 64822x + 633030	0.98	4.2−12.6
PS	5-hexene1, 3, 5-triyltribenzene	0.1−10 μg	y = 140908x + 13673	0.99	9.2−13.2
PE	1,12-tridecadiene	0.1−10 μg	y = 26634x + 23504	0.99	3.9−16.6
		10−200 μg	y = 15838x + 179704	0.99	10.6−16.9
PET	Vinyl benzoate	0.1−10 μg	y = 76077x + 16800	0.99	9.4−17.8
		10−200 μg	y = 53151x - 32925	0.98	4.4−17.8
PA	ε-caprolactam	0.1−10 μg	y = 269819x - 5550	0.99	5.9−16.9

Table S5. LOD and LOQ of different plastics at different sampling sites (μg/L).

Sampling site	LOD/LOQ (μg/L)					
	PMMA	PP	PS	PE	PET	PA
0	0.003/ 0.009	0.002/ 0.007	0.007/ 0.025	0.004/ 0.009	0.009/ 0.030	0.003/ 0.010
1	0.003/ 0.009	0.002/ 0.007	0.007/ 0.025	0.003/ 0.009	0.009/ 0.030	0.003/ 0.010
2	0.001/ 0.004	0.001/ 0.004	0.004/ 0.012	0.001/ 0.004	0.004/ 0.015	0.001/ 0.005
3	0.001/ 0.002	0.001/ 0.002	0.002/ 0.006	0.001/ 0.002	0.002/ 0.007	0.001/ 0.002

Table S6. The composition of plastic materials used in sampling and sample preparation.

Plastic materials	Plastic bucket	Ultrafiltration membrane	Connection tube	Centrifugal tube	Pipette head	Nitrile glove
Type	PE	PES	PE	PP	PP	Nitrile

Table S7. Recovery of selected MPs and NPs (PS).

Size range	Spiked con. (μg/L)	Tested con. (μg/L)			Recovery rate		
		PP	PS	PET	PP	PS	PET
100–400 μm	20	14.49 ± 0.96	15.08 ± 1.22	15.82 ± 1.19	72.43 ± 4.80	75.41 ± 6.10	79.08 ± 5.97
	10	6.68 ± 0.41	6.72 ± 0.21	7.14 ± 0.50	67.77 ± 4.15	67.17 ± 2.14	71.37 ± 5.04
	5	3.17 ± 0.53	3.55 ± 0.35	3.37 ± 0.42	63.40 ± 10.63	70.93 ± 7.02	67.40 ± 8.46
1–50 μm	20	13.90 ± 0.26	14.86 ± 0.59	14.77 ± 1.51	69.48 ± 1.31	74.32 ± 2.96	73.85 ± 7.56
	10	6.07 ± 0.41	6.48 ± 0.54	6.72 ± 0.46	60.73 ± 4.06	64.83 ± 5.39	67.17 ± 4.63
	5	3.21 ± 0.25	3.44 ± 0.12	3.27 ± 0.28	64.27 ± 4.99	68.73 ± 2.45	65.46 ± 5.66
0.02 μm	20	–	11.18 ± 1.23	–	–	55.88 ± 6.17	–
	10	–	5.45 ± 1.02	–	–	54.47 ± 10.21	–
	5	–	2.51 ± 0.44	–	–	50.13 ± 8.77	–

Table S8. Potential interferences of selected natural polymers (20 mg) with polymer indicator ions (before/after digestion).

	PVC Benzene	PMMA Methyl methacrylate	PP 2, 4-Dimethyl-1-heptene	PS 5-hexene1, 3, 5-triyltribenzene	PE 1,12-tridecadiene	PET Vinyl benzoate	PA ε-caprolactam
Wood	yes/no	no/no	no/no	no/no	no/no	no/no	no/no
leaf	yes/yes	no/no	no/no	no/no	no/no	no/no	no/no
fish	yes/?	no/no	no/no	no/no	no/no	no/no	no/no
Humic acid	yes/?	no/no	no/no	no/no	no/no	no/no	no/no
Black carbon (bamboo)	yes/yes	no/no	no/no	no/no	no/no	no/no	no/no

? = detected in traces, possible contamination.

Table S9. Mass concentrations of PVC, PMMA, PP, PS, PE, PET and PA (μg/L) measured in two WWTPs; ND: Not detectable.

Sampling site	Size Range (μm)	Mass concentration (μg/L)							
		PVC	PMMA	PP	PS	PE	PET	PA	Total
A-0	50−1000	7.663	0.368	9.274	0.705	3.730	7.19	2.166	39.322
		5.779	0.355	6.811	0.121	1.311	1.95	0.107	23.968
	1−50	3.700	0.376	3.300	0.008	0.546	1.982	1.113	11.314
		2.040	0.533	1.516	ND	0.506	1.257	0.567	5.977
	0.01−1	1.310	0.223	1.463	0.025	0.980	0.496	ND	3.575
		1.420	0.248	1.277	0.038	1.752	0.173	ND	3.310
A-1	50−1000	3.132	0.152	4.994	0.012	0.678	0.810	0.028	10.424
		1.375	0.120	3.263	0.030	0.824	0.987	0.051	5.998
	1−50	1.730	0.055	1.157	ND	0.166	0.864	ND	3.768
		1.841	0.080	0.962	ND	0.215	0.155	ND	3.561
	0.01−1	0.742	0.146	0.716	0.017	0.705	0.171	0.047	2.360
		0.513	0.134	0.573	0.018	0.285	0.533	0.049	1.542
A-2	50−1000	2.556	1.258	3.114	0.008	0.753	0.445	0.023	7.858
		2.687	0.341	4.308	ND	0.901	0.709	0.004	8.733
	1−50	0.065	0.047	0.446	ND	0.161	0.129	ND	0.894
		0.736	0.037	0.330	0.003	0.156	0.212	ND	1.268
	0.01−1	0.296	0.052	0.240	0.006	0.206	0.046	0.021	0.702
		0.201	0.131	0.660	ND	0.338	0.115	0.007	1.147
A-3	50−1000	0.343	0.073	0.766	ND	0.166	0.124	0.007	1.510
		0.201	0.093	0.643	ND	0.335	0.036	0.010	1.405
	1−50	0.111	0.008	0.297	ND	0.097	0.028	ND	0.518
		0.172	0.007	0.133	ND	0.137	0.071	ND	0.460
	0.01−1	0.071	0.006	0.107	0.004	0.066	0.021	ND	0.265
		0.054	0.006	0.168	ND	0.070	0.029	ND	0.283
B-0	50−1000	3.936	0.030	5.540	ND	0.729	1.154	0.008	11.687
		1.636	0.062	4.804	0.006	1.129	0.945	0.009	8.887
	1−50	0.468	ND	3.200	ND	0.365	0.282	ND	4.215
		0.074	0.028	2.126	ND	0.543	0.358	ND	3.029
	0.01−1	0.174	0.030	0.408	ND	0.207	0.045	ND	0.884
		0.296	0.028	0.364	ND	0.155	0.032	ND	0.835
B-1	50−1000	0.356	ND	1.662	0.028	ND	0.387	ND	2.506
		0.596	ND	0.912	0.006	0.943	0.367	ND	3.075
	1−50	0.838	0.028	1.460	ND	0.813	0.082	ND	3.161
		0.542	0.050	2.120	0.038	0.121	0.161	ND	2.932
	0.01−1	0.092	0.042	0.442	ND	0.265	0.069	ND	0.880
		0.072	ND	0.222	ND	0.177	0.041	ND	0.592
B-2	50−1000	0.112	0.098	0.680	0.014	ND	0.296	ND	1.200
		1.404	ND	0.790	0.022	0.563	0.146	ND	3.122
	1−50	0.334	ND	0.082	ND	ND	0.036	ND	0.450
		0.162	ND	0.730	0.006	ND	0.027	ND	0.937
	0.01−1	0.071	ND	0.242	ND	ND	0.014	ND	0.329
		0.045	ND	0.208	ND	ND	0.018	ND	0.271
B-3	50−1000	0.020	ND	0.274	ND	0.148	0.038	ND	0.512
		0.038	ND	0.224	0.006	0.109	0.043	ND	0.448
	1−50	0.056	ND	0.179	ND	ND	0.021	ND	0.249
		0.032	ND	0.090	ND	ND	0.016	ND	0.132
	0.01−1	0.036	ND	0.154	0.006	ND	0.009	0.008	0.215
		0.021	ND	0.084	0.006	ND	0.008	0.003	0.121

References

1. Li, Z.; Shakiba, S.; Deng, N.; Chen, J.; Louie, S. M.; Hu, Y., Natural Organic Matter (NOM) Imparts Molecular-Weight-Dependent Steric Stabilization or Electrostatic Destabilization to Ferrihydrite Nanoparticles. *Environ Sci Technol* **2020,** *54*, (11), 6761-6770.
2. McAdams, B. C.; Aiken, G. R.; McKnight, D. M.; Arnold, W. A.; Chin, Y. P., High Pressure Size Exclusion Chromatography (HPSEC) Determination of Dissolved Organic Matter Molecular Weight Revisited: Accounting for Changes in Stationary Phases, Analytical Standards, and Isolation Methods. *Environ Sci Technol* **2018,** *52*, (2), 722-730.
3. Tadanier, C. J.; Berry, D. F.; Knocke, W. R., Dissolved Organic Matter Apparent Molecular Weight Distribution and Number-Average Apparent Molecular Weight by Batch Ultrafiltration. *Environmental Science & Technology* **2000,** *34*, (11), 2348-2353.
4. Tian, J.; Wu, C.; Yu, H.; Gao, S.; Li, G.; Cui, F.; Qu, F., Applying ultraviolet/persulfate (UV/PS) pre-oxidation for controlling ultrafiltration membrane fouling by natural organic matter (NOM) in surface water. *Water Res* **2018,** *132*, 190-199.
5. Fischer, M.; Scholz-Bottcher, B. M., Simultaneous Trace Identification and Quantification of Common Types of Microplastics in Environmental Samples by Pyrolysis-Gas Chromatography-Mass Spectrometry. *Environ Sci Technol* **2017,** *51*, (9), 5052-5060.
6. Ribeiro, F.; Okoffo, E. D.; O'Brien, J. W.; Fraissinet-Tachet, S.; O'Brien, S.; Gallen, M.; Samanipour, S.; Kaserzon, S.; Mueller, J. F.; Galloway, T.; Thomas, K. V., Quantitative Analysis of Selected Plastics in High-Commercial-Value Australian Seafood by Pyrolysis Gas Chromatography Mass Spectrometry. *Environ Sci Technol* **2020,** *54*, (15), 9408-9417.
7. Zhou, X. X.; He, S.; Gao, Y.; Chi, H. Y.; Wang, D. J.; Li, Z. C.; Yan, B., Quantitative Analysis of Polystyrene and Poly(methyl methacrylate) Nanoplastics in Tissues of Aquatic Animals. *Environ Sci Technol* **2021,** *55*, (5), 3032-3040.
8. Blancho, F.; Davranche, M.; Hadri, H. E.; Grassl, B.; Gigault, J., Nanoplastics Identification in Complex Environmental Matrices: Strategies for Polystyrene and Polypropylene. *Environ Sci Technol* **2021,** *55*, (13), 8753-8759.
9. Nuelle, M. T.; Dekiff, J. H.; Remy, D.; Fries, E., A new analytical approach for monitoring microplastics in marine sediments. *Environ Pollut* **2014,** *184*, 161-9.
10. Okoffo, E. D.; Ribeiro, F.; O'Brien, J. W.; O'Brien, S.; Tscharke, B. J.; Gallen, M.; Samanipour, S.; Mueller, J. F.; Thomas, K. V., Identification and quantification of selected plastics in biosolids by pressurized liquid extraction combined with double-shot pyrolysis gas chromatography-mass spectrometry. *Sci Total Environ* **2020,** *715*, 136924.
11. Dumichen, E.; Eisentraut, P.; Bannick, C. G.; Barthel, A. K.; Senz, R.; Braun, U., Fast identification of microplastics in complex environmental samples by a thermal

degradation method. *Chemosphere* **2017,** *174*, 572-584.
12. Hermabessiere, L.; Himber, C.; Boricaud, B.; Kazour, M.; Amara, R.; Cassone, A. L.; Laurentie, M.; Paul-Pont, I.; Soudant, P.; Dehaut, A.; Duflos, G., Optimization, performance, and application of a pyrolysis-GC/MS method for the identification of microplastics. *Anal Bioanal Chem* **2018,** *410*, (25), 6663-6676.
13. Picó, Y.; Barceló, D., Pyrolysis gas chromatography-mass spectrometry in environmental analysis: Focus on organic matter and microplastics. *TrAC Trends in Analytical Chemistry* **2020,** *130*.
